# Supplementary material for: Compartmentalized Synthesis of Triacylglycerol at the Inner Nuclear Membrane Regulates Nuclear Organization
Source: Dev Cell. 2019 Sep 23;50(6):755–766.e6. doi: 10.1016/j.devcel.2019.07.009 (PMC6859503; doi:10.1016/j.devcel.2019.07.009)
Supplement: Document S1. Figures S1–S3 [file mmc1.pdf]

Developmental Cell, Volume *50*

Supplemental Information

Compartmentalized Synthesis of Triacylglycerol at the Inner Nuclear Membrane Regulates Nuclear Organization

Antonio D. Barbosa, Koini Lim, Muriel Mari, James R. Edgar, Lihi Gal, Peter Sterk, Benjamin J. Jenkins, Albert Koulman, David B. Savage, Maya Schuldiner, Fulvio Reggiori, Philip A. Wigge, and Symeon Siniossoglou

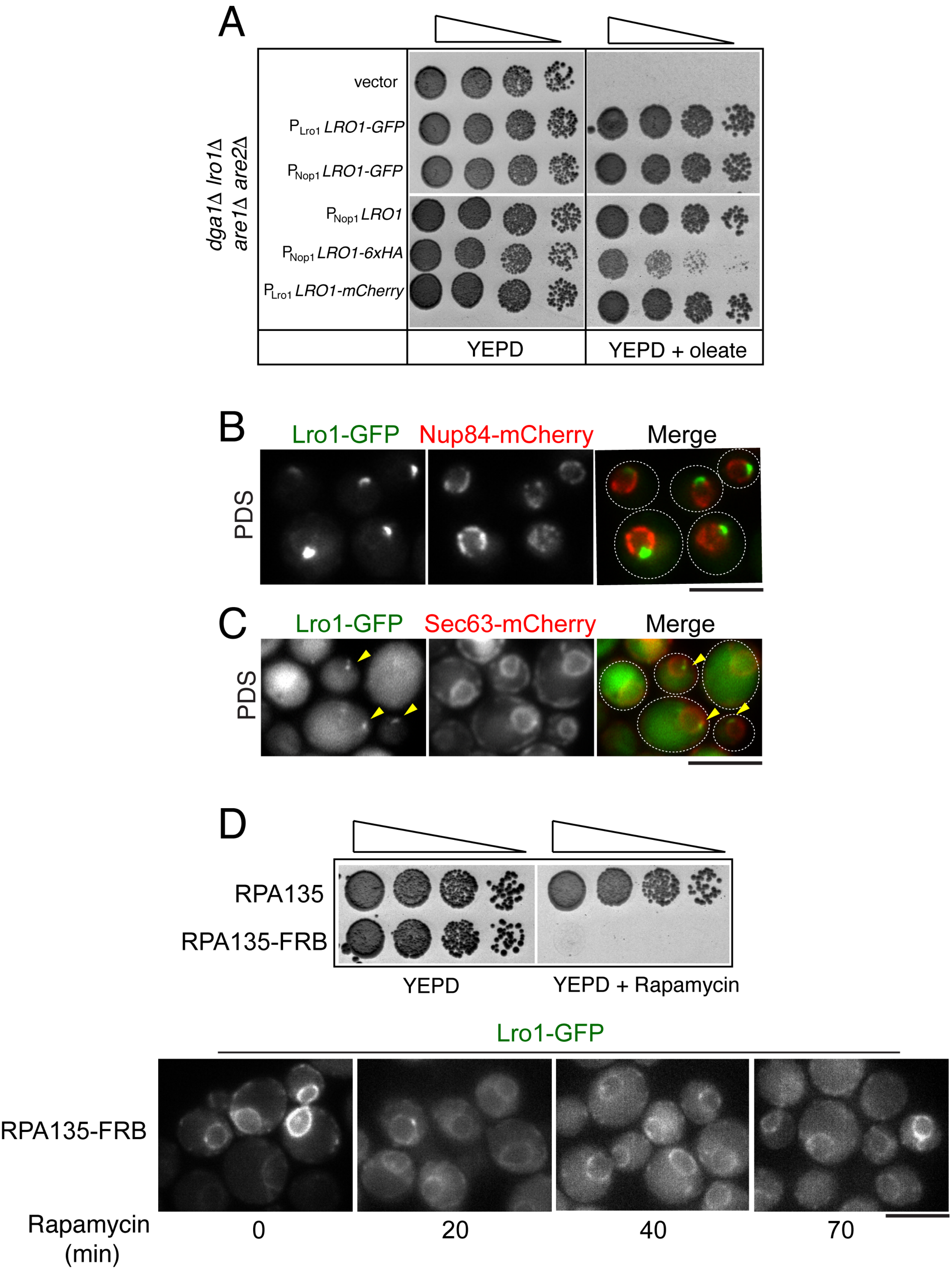

Figure S1. Related to Figure 1

Figure S1: Targeting of Lro1 to the nucleolar-associated membrane. (A) *dga1*Δ *lro1*Δ *are1*Δ *are2*Δ (4Δ) cells expressing the Lro1 epitope-tagged plasmids that were used in this study, were grown to the exponential phase and 0.2 $OD_{(600)}$ of cells were spotted on YEPD plates with or without oleate. 4Δ cells lack LDs and as a result lose viability when challenged with free fatty acid. Growth indicates a functional Lro1 fusion. P_{LRO1}; expression under the control of the *LRO1* promoter; P_{NOP1}; expression under the control of the *NOP1* promoter. (B) Co-localization of Lro1-GFP expressed under the control of the *NOP1* promoter, and the nuclear pore complex protein Nup84-mCherry in wild-type cells at the PDS phase. The outlines of cells are depicted. (C) Co-localization of Lro1-GFP integrated at its endogenous chromosomal locus and the ER reporter Sec63-mCherry in wild-type cells at the PDS phase. The arrowheads point to the Lro1-GFP punctum at the nuclear membrane. The outlines of cells are depicted. (D) Inhibition of rDNA transcription is not sufficient for stable targeting of Lro1 to the nucleolar-associated membrane. Top panel: the anchor away strain expressing an FRB-tagged version of the RNA PolI subunit RPA135 (strain SS2837), together with the parental untagged strain, were spotted on YEPD plates with or without rapamycin. Lower panel: the RPA135-FRB strain expressing Lro1-GFP were grown exponentially, rapamycin was added for the indicated times and cells were imaged live. Note that RNA PolI association with rDNA is reduced to 80% after 60 min rapamycin addition (Albert et al., 2016). Scale bars, 5 microns. Related to Figure 1.

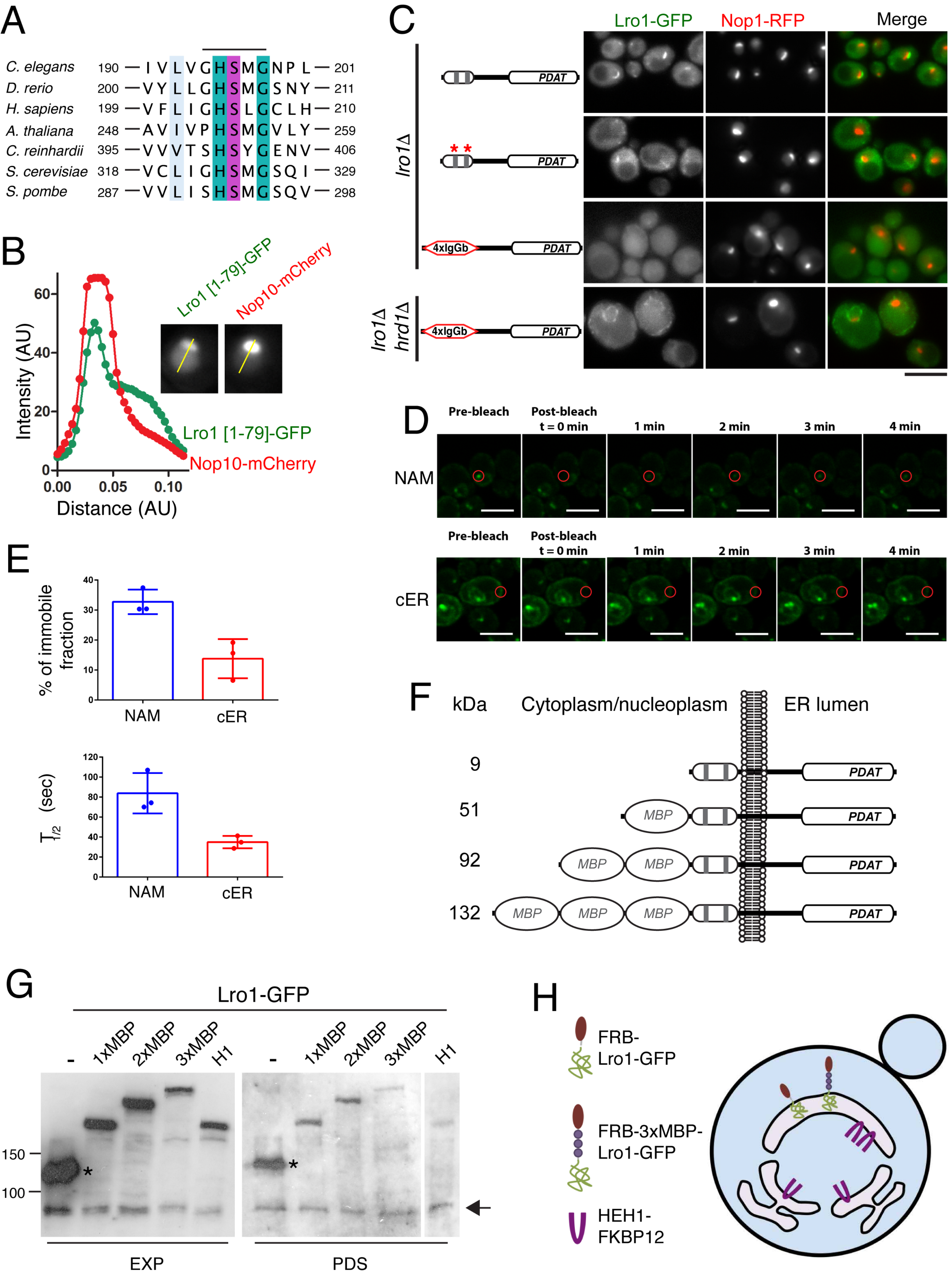

Figure S2. Related to Figure 2

Figure S2: Determinants and dynamics of Lro1 targeting to the inner nuclear membrane. (A) Alignment of the predicted catalytic GHSXG catalytic motif of Lro1 with other fungal, plant PDATs or metazoan LCATs that share a similar catalytic domain. (B) Pixel intensity profiles for Lro1[1-79]-GFP (green) and Nop10-mCherry (red) for the nucleus selected in the inset in Figure 2B; AU, arbitrary units. (C) Representative examples from the quantification shown in Figure 2C. (D) Typical FRAP of Lro1-GFP, expressed under the control of the *NOP1* promoter, at the nucleolar-associated membrane (NAM) or the cortical ER (cER). (E) Immobile fraction (top) and half-life (bottom) from measurements in 21 cells from three different experiments. (F) Schematic of the MBP fusions used; left column indicates the molecular mass (kDa) of the extralumenal domain appended. (G) A strain lacking all enzymes responsible for neutral lipid production (*dga1*Δ *lro1*Δ *are1*Δ *are2*Δ; 4Δ) was transformed with *CEN/URA3* plasmids expressing GFP fusions of *LRO1, LRO1-1xMBP -2xMBP, 3x-MBP and H1-LRO1*, all under the control of the *NOP1* promoter. Cells were grown to the exponential or PDS phases and equal amounts of cells were lysed and analysed (1.8 OD_{600} per lane) by western blot using an anti-GFP antibody. The star indicates the wild-type Lro1-GFP fusion in the exponential and PDS phases. The arrow denotes an antibody cross-reacting band. (H) Schematic of the anchor away assay used and the Lro1 fusions expressed; see text for details. Scale bars, 5 microns. Related to Figure 2.

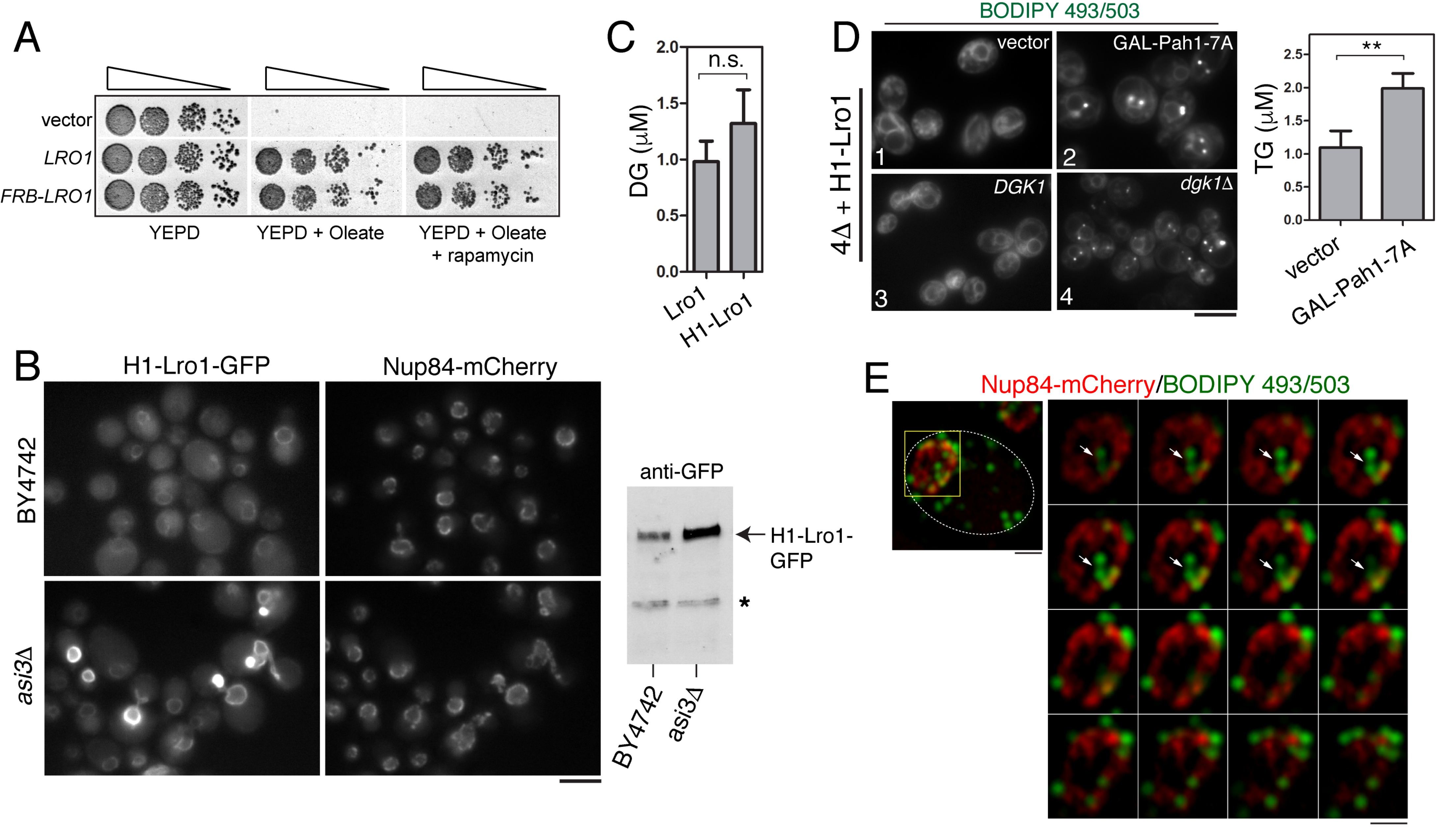

Figure S3. Related to Figures 4 and 5

Figure S3. Lro1 targeted constitutively at the INM is catalytically active. (A) A *dga1*Δ *lro1*Δ anchor away strain carrying the INM anchor (*HEH1-FKBP12*) and expressing the indicated fusions, were spotted on YEPD plates containing the denoted supplements. Cells were grown for two days at 30C. (B) H1-Lro1-GFP levels at the nuclear membrane increase in the *asi3*Δ mutant. Left panel: The indicated strains, expressing H1-Lro1-GFP and a Nup84-mCherry reporter, were grown to the PDS phase and imaged live. Right panel: samples from the same strains were lysed and analysed by western blot with an anti-GFP antibody as described under STAR Methods. The star denotes an antibody cross-reacting band. Scale bars: 5 microns. (C) Lipidomic quantification of cellular DG levels of the strains shown in Figure 5E, panels 1 and 2. DG levels shown are relative to internal DG standards of known concentration. Values are means from three independent cultures per strain. (D) 4Δ expressing H1-Lro1 and carrying an empty vector (panel 1) or the *GAL-PAH1-7A* plasmid (panel 2) were transferred to galactose-containing medium for 3 h and stained with BODIPY 493/503; right panel: samples from the cells shown in panels 1 and 2 were processed for lipidomic analysis as in C; values shown are means from three independent cultures per strain. Lower panels: exponentially growing 4Δ (panel 3) or 4Δ *dgk1*Δ (panel 4) cells expressing H1-Lro1 were stained with BODIPY 493/503. (E) 4Δ cells expressing Lro1 and Nup84-mCherry were grown to the PDS phase, stained with BODIPY 493/503, and imaged live using Zeiss LSM880 confocal microscope equipped with an Airyscan unit, as described in STAR Methods, at 0.18 µm axial resolution, and 0.2 µm step slices with 50% overlap. The arrow points to a representative intranuclear LD. Scale bars in B and D: 5 microns; in E: 1 micron. **, $p<0.01$; ns, not significant. Related to Figures 4 and 5.
