# Supplementary material for: Compartmentalized Synthesis of Triacylglycerol at the Inner Nuclear Membrane Regulates Nuclear Organization
Source: Dev Cell. 2019 Sep 23;50(6):755–766.e6. doi: 10.1016/j.devcel.2019.07.009 (PMC6859503; doi:10.1016/j.devcel.2019.07.009)
Supplement: Document S2. Article plus Supplemental Information [file mmc6.pdf]

Developmental Cell

Article

Compartmentalized Synthesis of Triacylglycerol at the Inner Nuclear Membrane Regulates Nuclear Organization

Graphical Abstract

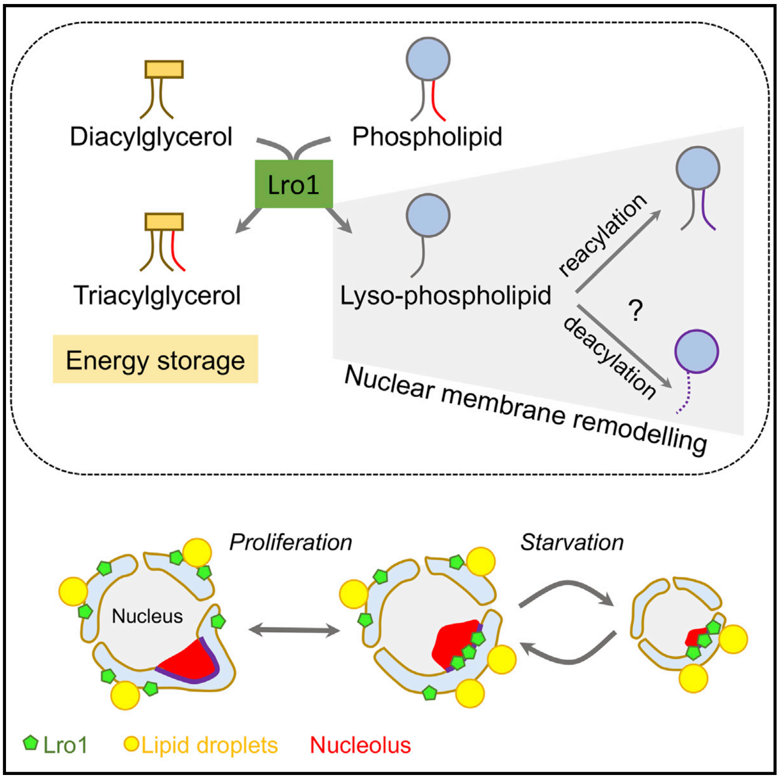

Authors

Antonio D. Barbosa, Koini Lim, Muriel Mari, ..., Fulvio Reggiori, Philip A. Wigge, Symeon Siniossoglou

Correspondence

ss560@cam.ac.uk

In Brief

Membrane phospholipids can be used as fatty acyl donors for the synthesis of triacylglycerols by enzymes called PDATs. Barbosa et al. present evidence that, in response to nutrient signals, the yeast PDAT Lro1 is active at the inner nuclear membrane, providing a link between organelle membrane remodeling and lipid storage.

Highlights

- Nutrients regulate the phospholipid:diacylglycerol acyltransferase Lro1
- Lro1 targets the INM subdomain bordering the nucleolus
- Lro1 is active at the INM and can sustain cell survival during starvation
- Compartmentalized synthesis of nuclear TG is important for nuclear envelope integrity

Barbosa et al., 2019, Developmental Cell *50*, 755–766
September 23, 2019 © 2019 The Authors. Published by Elsevier Inc.
https://doi.org/10.1016/j.devcel.2019.07.009

CellPress

Developmental Cell
Article

CellPress

Compartmentalized Synthesis of Triacylglycerol at the Inner Nuclear Membrane Regulates Nuclear Organization

Antonio D. Barbosa,[1] Koini Lim,[2] Muriel Mari,[3] James R. Edgar,[1] Lihi Gal,[4] Peter Sterk,[1] Benjamin J. Jenkins,[5] Albert Koulman,[5] David B. Savage,[2] Maya Schuldiner,[4] Fulvio Reggiori,[3] Philip A. Wigge,[6] and Symeon Siniossoglou[1,7,*]
[1]Cambridge Institute for Medical Research, University of Cambridge, Cambridge CB2 0XY, UK
[2]Metabolic Research Laboratories, Wellcome Trust-Medical Research, Council Institute of Metabolic Science, University of Cambridge, Cambridge CB2 0QQ, UK
[3]Department of Cell Biology, University of Groningen, University Medical Center Groningen, 9713AV Groningen, Netherlands
[4]Department of Molecular Genetics, Weizmann Institute of Science, Rehovot 7610001, Israel
[5]NIHR BRC Core Metabolomics and Lipidomics Laboratory and University of Cambridge Metabolic Research Laboratories, Wellcome Trust-Medical Research Council Institute of Metabolic Science, Cambridge CB2 0QQ, UK
[6]Sainsbury Laboratory, University of Cambridge, Cambridge CB2 1LR, UK
[7]Lead Contact
*Correspondence: ss560@cam.ac.uk
https://doi.org/10.1016/j.devcel.2019.07.009

SUMMARY

Cells dynamically adjust organelle organization in response to growth and environmental cues. This requires regulation of synthesis of phospholipids, the building blocks of organelle membranes, or remodeling of their fatty-acyl (FA) composition. FAs are also the main components of triacyglycerols (TGs), which enable energy storage in lipid droplets. How cells coordinate FA metabolism with organelle biogenesis during cell growth remains unclear. Here, we show that Lro1, an acyltransferase that generates TGs from phospholipid-derived FAs in yeast, relocates from the endoplasmic reticulum to a subdomain of the inner nuclear membrane. Lro1 nuclear targeting is regulated by cell cycle and nutrient starvation signals and is inhibited when the nucleus expands. Lro1 is active at this nuclear subdomain, and its compartmentalization is critical for nuclear integrity. These data suggest that Lro1 nuclear targeting provides a site of TG synthesis, which is coupled with nuclear membrane remodeling.

INTRODUCTION

The internal membrane systems of the eukaryotic cell are highly dynamic, and their regulated remodeling is essential for proper organelle function, during both normal cell growth and stress. For example, the nuclear membrane undergoes dynamic remodelling during the two modes of nuclear division operating in eukaryotes, “open” and “closed” mitosis (Zhang and Oliferenko, 2013; Ungricht and Kutay, 2017), and the accumulation of unfolded proteins in the endoplasmic reticulum (ER) drives significant ER membrane expansion in order to support increased protein folding capacity (Walter and Ron, 2011). Therefore, cells must possess mechanisms to selectively add, remove, or remodel membrane phospholipids at different organelles in response to cell cycle and stress signals, but those remain poorly understood.

Lipid precursors, which are normally directed toward membrane synthesis to promote cell growth, are diverted toward storage during nutrient limitation. The main metabolic energy storage molecules in eukaryotes are triacylglycerols (TGs) which, together with other neutral lipids (e.g., steryl esters), are deposited in lipid droplets (LDs) (Wang et al., 2017). LDs emerge from, and remain associated with, the ER membrane in many cell types and interact with other organelles (Barbosa and Siniossoglou, 2017). Some of these interactions involve LDs with their “client” organelles, such as mitochondria and peroxisomes, which catabolize fatty acids stored in TGs and provide an essential source of energy during starvation (Herms et al., 2015; Rambold et al., 2015). Other interactions suggest a link of LDs to organelle membrane biogenesis. For example, LDs have been proposed to provide lipid precursors for autophagosome membrane biogenesis in yeast and mammals (Dupont et al., 2014; Shpilka et al., 2015). Similarly, a specific pool of LDs associate with the expanding prospore membrane that sequesters the meiotic nuclei during sporulation of yeast cells (Ren et al., 2014; Hsu et al., 2017), and LD-mobilized fatty acids are required for bud growth and cell cycle progression in yeast (Kurat et al., 2009).

TG is synthesized by acyl-CoA:diacylglycerol acyltransferases (DGATs) or phospholipid-diacylglycerol acyltransferases (PDATs) (Ruggles et al., 2013). Most eukaryotes express DGATs while PDATs have been described so far in fungi, microalgae, and plants. Whereas DGATs use a fatty acid activated with coenzyme A (FA-CoA) to acylate diacylglycerol (DG), PDATs transfer a fatty acid from a phospholipid directly to DG (Figure 1A). Accordingly, PDATs couple TG synthesis to membrane phospholipid deacylation (Dahlqvist et al., 2000; Oelkers et al., 2000) (Figure 1B).

Here, we uncover a phospholipid remodeling pathway that targets a specific subdomain of the inner nuclear membrane (INM). We find that the PDAT Lro1 of *Saccharomyces cerevisiae* (from hereon called yeast) is imported from the ER to the INM

This is an open access article under the CC BY license (http://creativecommons.org/licenses/by/4.0/).

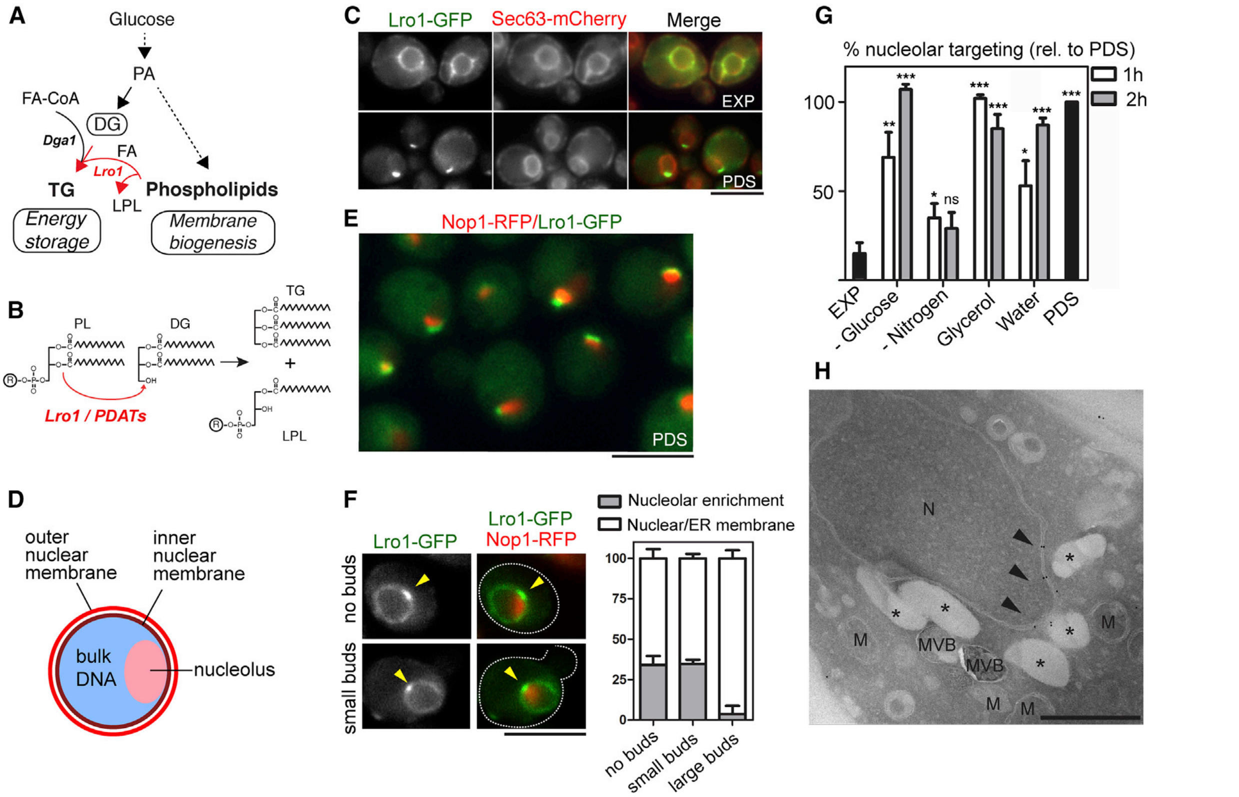

Figure 1. Lro1 Targets a Nuclear Membrane Subdomain that Associates with the Nucleolus

(A) Schematic of the major lipid metabolic pathways in yeast; PA, phosphatidate; DG, diacylglycerol; TG, triacylglycerol; FA, fatty acid: LPL, lysophospholipid.

(B) Schematic of the PDAT activity; PL, phospholipid.

(C) Localization of Lro1-GFP expressed under the control of its own promoter in cells co-expressing an ER (Sec63-mCherry) reporter at the indicated growth phases.

(D) Schematic of the organization of the yeast nucleus.

(E) Co-localization of Lro1-GFP as in C but with a nucleolar reporter.

(F) Left panels: examples of nucleolar enrichment of Lro1-GFP during the exponential phase; right panel, quantification from three experiments, n = 343 cells.

(G) Quantification of Lro1 targeting to the nucleolar-associated membrane in response to various stresses. Exponentially growing cells expressing a chromosomally integrated nucleolar reporter (*NOP10*-mCherry) were subjected to the indicated stresses and the percentage of Lro1-GFP targeting to the nucleolar-associated membrane was quantified (n = 3 experiments, at least 600 cells counted per stress condition); comparisons are between 1 or 2 h and PDS.

(H) Immunolabeling of chemically fixed yeast cells expressing Lro1-6xHA. Arrowheads point to gold particles clustering on one side of the nuclear envelope. Stars indicate LDs. MVB, multivesicular bodies; M, mitochondria. Scale bars in (C), (E), and (F), 5 μm; in H, 500 nm. $^{*}p < 0.05$; $^{**}p < 0.01$; $^{***}p < 0.001$; ns, not significant. See also Figure S1.

abutting the nucleolus. Lro1 is active at this specific nuclear subdomain resulting in the utilization of phospholipid-derived fatty acids to generate TGs and lysophospholipids. Interestingly, targeting of Lro1 is regulated by cell cycle and nutrient signals and is inhibited when the nucleus expands. Notably, we find that synthesis of TG at the INM sustains survival during starvation, suggesting the presence of a pathway that exports TG to the cytoplasmic side of the ER.

RESULTS

Cell Cycle and Nutrient Signals Cause Dynamic Targeting of Lro1 to a Nuclear Membrane Subdomain Associated with the Nucleolus

To determine if PDATs have a role in specific membrane remodeling events during nutrient depletion, we examined the subcellular localization of a C-terminally GFP-tagged Lro1 fusion protein when nutrients start to become scarce. All Lro1 fusions used for localization studies were catalytically active (Figure S1A). Lro1-GFP localizes to the ER during the exponential growth phase (EXP), when lipid intermediates are used to drive phospholipid synthesis to sustain rapid growth, but it relocates to a subdomain of the nuclear envelope as cells face nutrient depletion during diauxic shift (post-diauxic shift [PDS] phase; Figure 1C; Wang and Lee, 2012). This was observed when plasmid-borne Lro1-GFP was expressed from its own promoter or from the stronger *NOP1* promoter (Figures 1C and S1B) as well as when Lro1-GFP was integrated at its chromosomal locus (Figure S1C). The morphology of the Lro1-GFP membrane domain is reminiscent of the nucleolus, which adopts a crescent-like shape and is tethered to the INM in yeast (Taddei and Gasser, 2012) (Figure 1D). Using the nucleolar reporter

Nop1-RFP, we demonstrated that Lro1-GFP indeed accumulates at the membrane bordering the nucleolus (Figure 1E). Interestingly, careful analysis of Lro1 localization during exponential phase also revealed, in addition to its ER localization, an enrichment of Lro1 at the subdomain bordering the nucleolus in 34.0% ± 5.6% unbudded and 34.5% ± 2.7% small budded cells, but only in 3.8% ± 5.0% of large budded cells (Figure 1F). This is consistent with Lro1-GFP accumulation at the nucleolus in PDS phase since yeast cells arrest at the G1 phase of the cell cycle at the diauxic shift (Miles et al., 2013). We also observed a similar accumulation of Lro1-GFP at this subdomain during acute glucose starvation, during growth in non-fermentable carbon sources, or when transferring the cells in water but not upon nitrogen deprivation (Figure 1G) or inhibition of rDNA transcription (Figure S1D). Immunoelectron microscopy revealed that an Lro1-6xHA fusion preferentially associated with the perinuclear ER during exponential phase (i.e., 62.6% ± 0.36%), and only part of it was at the cortical and/or peripheral ER (37.3% ± 0.21%), respectively. In the PDS phase, a significant decrease in Lro1 protein levels (see later) reduced the labeling efficiency, precluding statistical quantifications. Nevertheless, in the few cell sections where Lro1-6xHA was detected, this fusion protein was mostly found on one side of the nuclear envelope and always adjacent to LDs (Figure 1H). Taken together, these results show that glucose limitation and cell cycle signals target Lro1 to a subdomain of the nuclear membrane, which is in contact with the nucleolus.

Lro1 Is Targeted to the INM

Lro1 is a type II integral membrane protein with a short basic cytosolic N-terminal domain and a larger luminal catalytic domain (Figures 2A and S2A; Choudhary et al., 2011). Expression of its N-domain fused to GFP shows a clear intranuclear localization with enrichment at the nucleolus (Figures 2B, panel 2 and S2B). Notably, the N-domain with the transmembrane segment also accumulates at the membrane in contact with the nucleolus (Figure 2B, panel 4 versus 6). Mutating the K/R residues within two basic clusters into alanines abrogates the nucleolar enrichment of both fusions in PDS phase (Figure 2B, panel 2 versus 3; panel 4 versus 5). Unexpectedly, the same mutations only partially compromise the targeting of Lro1 within the context of the full-length protein, indicating the presence of additional targeting determinants (Figures 2C and S2C). To examine whether these also map in the N-domain, we replaced it with 4 IgG binding domains of Protein A (4xIgGb) and found that this prevented detection of the resulting GFP fusion at the PDS phase, both at the nucleolus and at the ER. Because the stability of Lro1 is controlled by the ubiquitin-protein ligase Hrd1 (Iwasa et al., 2016), we imaged 4xIgGb-Lro1-GFP in an *hrd1*Δ strain and found out that it could be detected at the ER but not at the nucleolar-associated membrane, at the PDS phase (Figures 2C and S2C). Together, these results show that the N-domain of Lro1 is necessary and sufficient for its efficient targeting to the nuclear membrane subdomain in contact with the nucleolus; when this targeting fails in PDS, Lro1 is unstable in the ER.

Next, we compared the dynamics of the two pools of Lro1 using fluorescence recovery after photobleaching (FRAP). We found that these exhibit different properties: the nucleolar-associated membrane pool of Lro1-GFP was more immobile and its fluorescence recovery was significantly slower than that of the cortical ER, suggesting the presence of a limiting step in its targeting (Figures 2D, S2D, and S2E). Given that its soluble N-domain associates to the nucleolus, we asked whether Lro1 accesses the inner side of the nuclear membrane. We applied two assays to address this question: the first approach was based on the fact that import of integral membrane proteins from the ER to the INM through the nuclear pore is limited by the size of their cytosolic domains, with the cutoff in yeast being 90 kDa (Popken et al., 2015). In support of Lro1 residing in the INM, Lro1-GFP nucleolar targeting was significantly compromised when its extralumenal domain was made larger by appending 1, 2, or 3 copies of the maltose-binding protein (MBP) (Figures 2E and S2F). Moreover, we found that increasing the size of the N-terminal domain of Lro1 and/or preventing its nuclear import results in a significant decrease in its protein levels (Figure S2G), explaining why many cells have low 3xMBP-Lro1-GFP signal at the ER in the PDS phase. To independently determine whether Lro1 can associate with the INM, we used a second assay based on the anchor-away technique (Haruki et al., 2008). This approach requires the co-expression of two chimeric proteins: firstly, the INM protein Heh1 was fused to the FK506 binding protein (FKBP12); secondly, Lro1-GFP, or GFP as control, was fused to the FKBP12-rapamycin-binding (FRB) domain (Figure S2H). FRB and FKBP12 form a high-affinity ternary complex in the presence of rapamycin if they are in close proximity. Following the addition of rapamycin, FRB-GFP changed rapidly (30 min) from a diffuse to a ring-like localization, confirming that the INM anchor is indeed accessible to FRB-GFP (Figure 2F). Addition of rapamycin in the strain expressing FRB-Lro1-GFP resulted in the loss of its cortical ER localization and its accumulation at a perinuclear ring, which is typical of INM proteins. In contrast, FRB-3xMBP-Lro1-GFP retained its cortical ER localization after rapamycin treatment (Figure 2F), consistent with an impairment in nucleolar targeting when the mass of the N-terminal domain increases. Taken together, these data show that Lro1 targets the INM by virtue of its N-domain.

Lro1 Is Catalytically Active at the INM in Contact with the Nucleolus

The nuclear membrane associated with the nucleolus has the property of being particularly susceptible to expansion in response to excess phospholipid synthesis (Campbell et al., 2006; Karanasios et al., 2010; Witkin et al., 2012). We therefore examined whether Lro1 is active by following TG synthesis at this membrane subdomain. To do this, we first sought to express Lro1 in a background where it would be the sole source of neutral lipid; hence, we used a mutant with deletions in the two DG acyltransferases (*LRO1* and *DGA1*) and the two steryl acyltransferases (*ARE1* and *ARE2*), henceforth called 4Δ, and which lacks neutral lipids and LDs (Oelkers et al., 2002; Petschnigg et al., 2009). Given that synthesis of neutral lipids is essential for cell survival in stationary phase, 4Δ cells display accelerated cell death in PDS that can be rescued by the expression of Lro1 as the only source of TG. This rescue requires the catalytic activity of Lro1 because mutation of Ser324 within its conserved GHSXG lipase motif abolishes the appearance of LDs (data not shown) and survival in PDS (Figure 3A). Importantly, TG levels rise concomitantly with increases in the levels of Lro1-GFP at the

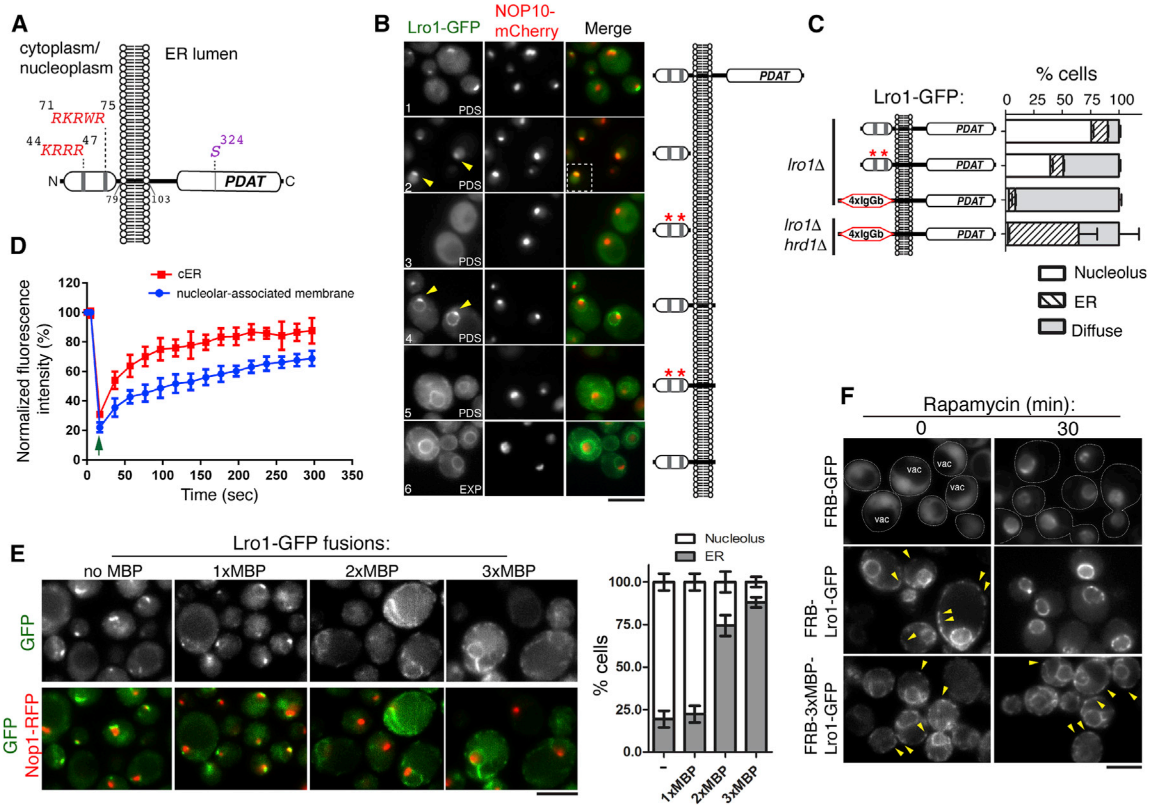

Figure 2. Translocation of Lro1 to the INM that Associates with the Nucleolus

(A) Schematic of the topology of Lro1. The K/R-rich nucleolar targeting sequences are shown in red. The Ser324 within the GHSXG lipase motif is shown.

(B) *lro1*Δ cells expressing a nucleolar reporter and the Lro1-GFP mutants shown were imaged at the indicated growth phases. Red stars denote the K/R to A mutations. Arrowheads denote the nucleolus and/or the nucleolar-associated membrane.

(C) Quantification of the subcellular localization of the indicated Lro1-GFP mutants in the specified strains. Red stars denote the K/R to A mutations within the extralumenal domain. Three colonies of each strain were analyzed; at least 200 cells were counted for each strain.

(D) Lro1-GFP was photobleached, either at the nucleolar-associated membrane or the cortical ER (cER), and fluorescence recovery was measured. Data are means ± SD from three independent experiments (seven cells each); arrow indicates the bleaching event.

(E) Nucleolar-associated membrane targeting of 1x-, 2x-, or 3x-MBP-Lro1-GFP fusions during the PDS phase. Right panel: Quantification of the data shown from three experiments, counting only cells with signal in ER or nucleolus; at least 250 cells were quantified for each strain.

(F) Localization of the FRB-GFP control (the outlines of cells are shown; vac, vacuole), and the FRB-Lro1-GFP (middle) or FRB-3xMBP-Lro1-GFP (bottom) fusions, before or after the addition of rapamycin. Arrowheads point to the cortical ER membrane. Scale bars in all micrographs, 5 μm. See also Figure S2.

nucleolar-associated membrane in 4Δ cells in time course experiments during exit from exponential growth (Figures 3B and 3C). Three-dimensional reconstruction of the LD distribution relative to Lro1-mCherry in 4Δ cells shows that in 86% (±2.5%, n = 3 experiments) of the cells at least one LD is in close proximity with the Lro1-mCherry punctum in the PDS phase (Figure 3D; Video S1). LDs in the vicinity of the nucleolus were also observed during live imaging of 4Δ cells expressing Lro1 (Video S2). In both experiments, LDs that are not in proximity to the nucleolus can still be detected; these may be derived from Lro1 activity at the ER during the exponential phase or their mobility within the perinuclear ER. Taken together, these results support the notion of Lro1 being active at the INM.

Lro1 Activity Regulates Phospholipid Homeostasis

Next, we investigated the effect of Lro1 activity on membrane phospholipid homeostasis. We hypothesized two scenarios: in the first, the lysophospholipid generated by Lro1 could be reacylated with a different fatty acid, changing the physical properties of the membrane; in the second, the lysophospholipid could be further broken down by a phospholipase, effectively degrading the original phospholipid substrate of Lro1 (Figure 3E). To discriminate between these possibilities, we first determined the subcellular distribution of the known lysophospholipid acyltransferases (Ale1 and Slc1) or phospholipases B (Nte1 and Lpl1; Plb's 1 to 3 could not be visualized) when Lro1 localizes to the nucleolar-associated membrane. GFP-fusions of Ale1 and Nte1, and to a lesser degree Slc1, localized to the ER with no apparent co-enrichment with Lro1-mCherry during the PDS phase (Figure 3F). Next, we determined the consequences of Lro1 activity in lipid homeostasis: consistent with its PDAT activity, we found that transient overexpression of Lro1 in wild-type cells caused an increase in TG levels (Figure 3G). Cells lacking Ale1, which has general lysophospholipid acyltransferase activity in yeast (Jain et al., 2007; Riekhof et al., 2007; Tamaki et al., 2007), showed an increase in both lyso-PC and lyso-PE levels

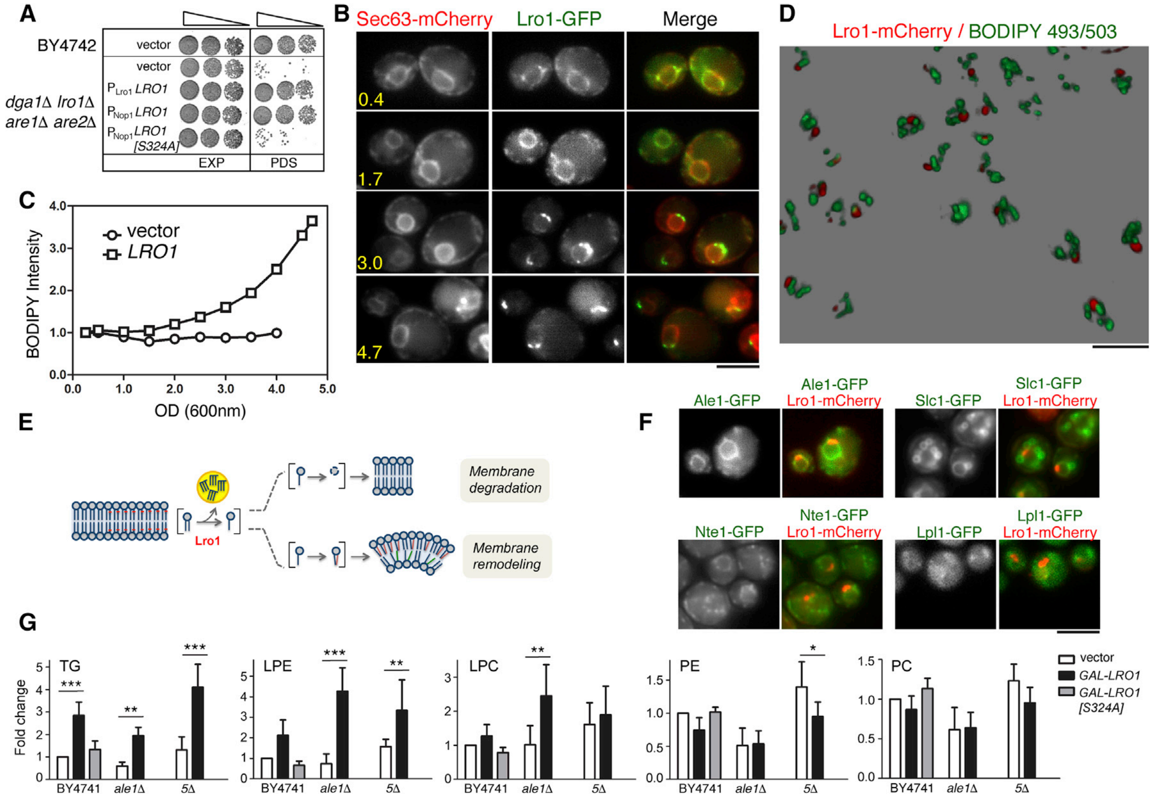

Figure 3. Lro1 Is Catalytically Active at the Nucleolar-Associated INM

(A) Wild-type or *dga1*Δ *lro1*Δ *are1*Δ *are2*Δ (4Δ) cells expressing the indicated plasmids were grown to exponential (EXP) or PDS phases in minimal synthetic medium and spotted on YEPD plates.

(B) 4Δ cells expressing Lro1-GFP and Sec63-mCherry were grown from exponential phase to the indicated densities and imaged.

(C) 4Δ cells expressing Lro1, or an empty vector, were grown to the indicated densities, labeled with BODIPY 493/503 and their fluorescence was quantified by FACS. Data are representative of two independent experiments.

(D) 4Δ cells expressing Lro1-mCherry were grown to the PDS phase and labeled with BODIPY 493/503. Deconvolved through-focus image series were processed to generate 3D image. The full reconstructed field is shown in Video S1.

(E) Model for the Lro1-mediated regulation of phospholipid homeostasis; see text for details.

(F) Co-localization of the indicated GFP fusions with Lro1-mCherry at the PDS phase.

(G) Lipidomic quantifications of TG, LPE, LPC, PE, and PC in wild-type (BY4741), ale1Δ, and *plb1*Δ *plb2*Δ *plb3*Δ *nte1*Δ *lro1*Δ (5Δ) cells expressing the denoted plasmids. Cells were grown in galactose for 5 h. Lipid levels were normalized to the corresponding levels of the wild-type strain expressing the empty vector. Data shown are means of at least 5 experiments ± SD. *p < 0.05; **p < 0.01; ***p < 0.001. Scale bars in all micrographs, 5 μm. See also Videos S1 and S2.

compared with that seen in the wild-type strain under the same conditions; on the other hand, a mutant strain lacking four known phospholipases B showed no change in lyso-PC and a more modest increase in lyso-PE compared to that of the *ale1*Δ mutant (Figure 3G). This result is consistent with the Lro1-derived lysophospholipids being directed primarily to re-acylation. However, our data cannot exclude a role for additional enzymes in the processing of Lro1-derived lysophospholipids.

Lro1 Localization Correlates with Changes in Nuclear Morphology

Next, we sought to investigate the nuclear function of Lro1 by mutating proteins required for its INM targeting. To do this, we performed an unbiased screen for factors involved in Lro1 nucleolar targeting. We used high content automated microscopy, followed by manual inspection, to determine the localization of Lro1-GFP in 6,000 strains carrying the loss of function mutations in all yeast genes. We focused on mutants with defects in Lro1 targeting during the PDS phase (and hence displaying increased ER localization compared to the wild-type) or decreased overall Lro1-GFP signal due to the degradation of its ER pool in the PDS phase. We identified 137 such mutants, which affect diverse cellular functions (Table S1). Nearly half of the genes of the ontology term "establishment of sister chromatid cohesion" affected Lro1-GFP targeting (Table S1). These included components of the Ctf19 complex of the kinetochore, which display a G2/M delay, consistent with the finding that Lro1 localization is regulated during the cell cycle. In those mutants, Lro1-GFP showed increased ER localization mostly in large budded cells (Figure 4A).

During G2/M mitotic delay, phospholipid synthesis is not halted resulting in expansion of the nuclear membrane that contains the nucleolus (Witkin et al., 2012). We asked whether other conditions that result in the expansion of this membrane domain correlate with loss of Lro1 targeting. During exposure to α-factor mating pheromone, a MAP kinase cascade induces

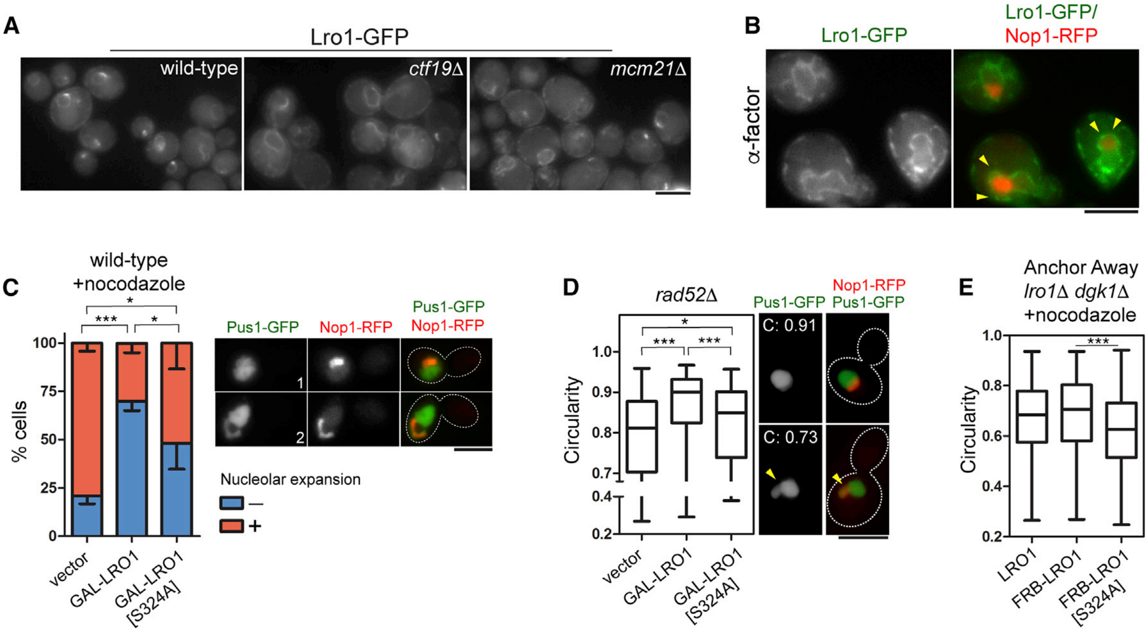

Figure 4. Effects of Lro1 on Nuclear Morphology

(A) Lro1-GFP localization in BY4742 (wild-type), *ctf19Δ*, and *mcm21Δ* strains grown to the PDS phase.

(B) The BY4741 strain expressing the indicated protein fusions was treated with α-factor; arrowheads point to the nuclear envelope "pocket" that encompasses the nucleolus.

(C) BY4741cells expressing *PUS1-GFP*, *NOP1-RFP*, and an empty vector or a high-copy *GAL-LRO1* plasmid were transferred to galactose-containing medium to induce *LRO1* expression, incubated with nocodazole, and extended focal images were collected live. The percentage of arrested cells displaying the elongated nuclear membrane expansion containing the nucleolus (panel 2) was determined; panels 1 shows a typical nucleus without membrane expansion; data shown are means of 5 experiments (at least 360 cells per strain) ± SD.

(D) *rad52Δ* cells expressing the indicated fluorescent fusion proteins and the denoted *LRO1* plasmids, were grown at the exponential phase and imaged as above; nuclear circularity of large budded cells was obtained from extended focal images cells as described in STAR Methods; right panels depict circularity measurements from round or expanded *rad52Δ* nuclei; arrowheads point to the nucleolar-associated membrane expansion; data shown are means of 6 experiments (at least 360 cells per strain) ± SD.

(E) A strain carrying an INM anchor (see Figure 2) and expressing *PUS1-mCherry* and the indicated Lro1 fusions were incubated first with rapamycin, followed by nocodazole. Nuclear circularity was calculated as in D; data shown are means of 3 experiments (at least 260 cells per strain) ± SD. Scale bar for all micrographs, 5 μm. *p < 0.05; ***p < 0.001. See also Figure S3.

the formation of an extended nuclear membrane "pocket" embracing the nucleolus (Stone et al., 2000) (Figure 4B). Lro1-GFP is excluded from this subdomain in α-factor treated cells, while it was still detected in contact with the nucleolus in unperturbed cycling G1/S cells. Thus, under multiple conditions where the nucleus expands, cycling anaphase (Figure 1F), G2/M delayed (Figure 4A) or α-factor-treated cells (Figure 4B), Lro1-GFP is displaced from the nucleolar-associated membrane.

The exclusion of Lro1 from the expanding INM suggests that its presence could modulate nuclear morphology. To test this, we transiently overexpressed Lro1 under two conditions that promote nucleolar membrane expansion (Witkin et al., 2012). Firstly, galactose-driven expression of Lro1 led to a 2.6-fold decrease in the percentage of wild-type cells with expanded nuclei following G2/M arrest with nocodazole (from 79% ± 4% to 30% ± 5%, $p < 0.001$) (Figure 4C). Secondly, we measured nuclear circularity in large budded *rad52Δ*, which display nucleolar expansion (Witkin et al., 2012), following galactose-induced Lro1 expression. Circularity ranges from 0 to 1, the latter corresponding to a perfect circle. *rad52Δ* nuclear circularity increased following Lro1 overexpression (from 0.78 ± 0.14 to 0.86 ± 0.12, $p < 0.001$). Although Lro1-S324A overexpression also led to an increase in nuclear circularity in both conditions, the catalytic active enzyme was more efficient in restoring nuclear shape (Figures 4C and 4D). We next examined the role of Lro1 at the INM using the anchor-away technique in cells expressing the INM anchor Heh1-FKBP12 in combination with Lro1, FRB-Lro1, or FRB-Lro1-S324A. We first confirmed that FRB-Lro1 is catalytically active when anchored at the INM (Figure S3A). Next, we incubated the cells with rapamycin to anchor the FRB-Lro1 fusions to the INM and then induced nuclear expansion with nocodazole. Since our data show that Lro1 is controlled through both targeting and DG availability (see later), we deleted *DGK1* in this system to increase the DG levels at the INM. We find that FRB-Lro1 activity at the INM is required to increase nuclear circularity (Figure 4E). Collectively, these data show that Lro1 localization correlates with, and impacts, the membrane expansion of this nuclear subdomain.

Nuclear TG Synthesis Is Sufficient to Sustain Growth during Starvation

Our data are consistent with a role for Lro1 in TG synthesis at the INM. However, since Lro1 partitions dynamically between cortical and perinuclear ER, it is possible that a pool of Lro1

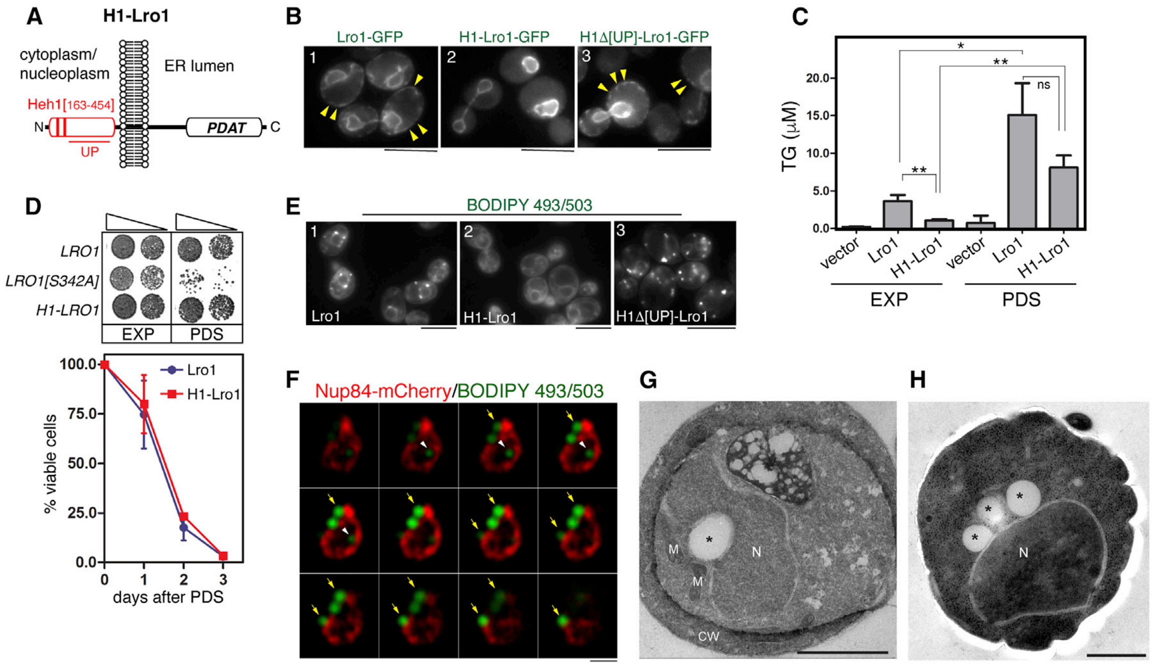

Figure 5. INM Activity of Lro1 Supports TG Synthesis and Is Induced by Availability of Diacylglycerol

(A) Schematic of the H1-Lro1 fusion. The Heh1 residues fused to Lro1 are shown; UP, unfolded peptide sequence.

(B) Localization of the denoted Lro1-GFP fusions in 4Δ cells. Arrowheads point to the cortical ER membrane.

(C) The 4Δ strain expressing either Lro1 or H1-Lro1, or an empty plasmid, was grown to the exponential or PDS phases. Lipids were extracted and TG quantified by mass spectrometry. TG levels shown are relative to internal TG standards of known concentration. Values shown are means from three independent cultures per strain.

(D) Upper panel: growth of 4Δ cells in minimal synthetic medium expressing wild-type Lro1, or the indicated Lro1 mutants, in exponential phase or following recovery from the PDS phase. 5-fold dilutions were spotted onto YEPD plates. Lower panel: Survival of 4Δ cells expressing Lro1, or H1-Lro1, in minimal medium. Data are means ± SDs from three different cultures per strain.

(E) Exponentially growing 4Δ cells expressing the indicated Lro1 constructs were stained with BODIPY 493/503 to label LDs.

(F) 4Δ cells expressing H1-Lro1 and Nup84-mCherry were grown to the PDS phase, stained with BODIPY 493/503, and imaged live using Zeiss LSM880 confocal microscope equipped with an Airyscan unit, as described in STAR Methods, at 0.18 μm axial resolution, and 0.2 μm step slices with 50% overlap. The arrowhead points to a representative intranuclear LD. Arrows point to LDs that associate with the outer nuclear membrane.

(G) 4Δ cells expressing H1-Lro1 were grown to the PDS phase and processed for electron microscopy as described in STAR Methods. CW, cell wall; M, mitochondria; N, nucleus; LD is marked with an asterisk.

(H) 4Δ cells expressing H1-Lro1 were grown to the PDS phase and processed for high-pressure freezing and freeze substitution as described in STAR Methods. Scale bars in (B) and (E), 5 μm; in (F) and (G), 1 μm; in (H), 500 nm. *$p < 0.05$; **$p < 0.01$; ns, not significant.

See also Figure S3.

remains active at the cortical ER at the PDS phase. Therefore, we asked whether redirecting Lro1 constitutively to the INM would be sufficient to support TG synthesis. We found that the INM-targeting sequence of Heh1 (Meinema et al., 2011) is sufficient to localize an Lro1 fusion (H1-Lro1-GFP) exclusively to a perinuclear ring both in wild-type or 4Δ cells, which is indicative of INM targeting (Figures 5A and 5B, panels 1 and 2). Consistently, disruption the Asi ubiquitin ligase complex, which mediates INM protein-specific degradation (Foresti et al., 2014; Khmelinskii et al., 2014), led to an increase in both the protein and the nuclear membrane fluorescence levels of H1-Lro1-GFP (Figure S3B). Two lines of evidence indicate that H1-Lro1 is active at generating TG at the INM in PDS phase: firstly, expression of H1-Lro1 in 4Δ led to a significant increase of TG levels (Figure 5C) and secondly, H1-Lro1 rescued the lethality of 4Δ in the PDS phase (Figure 5D). Strikingly, TG synthesis at the INM did not compromise long-term survival in stationary phase (Figure 5D).

We noticed, however, that 4Δ H1-Lro1 cells lack detectable LDs during exponential growth in standard glucose media (Figure 5E, panels 1 and 2). Cellular levels of DG, which is required for the formation of LDs, do not decrease significantly in the H1-Lro1 strain in the exponential phase (Figure S3C). We therefore hypothesize that the INM is less accessible to DG than the cytosolic ER during the exponential phase. Two results support this hypothesis: (1) re-localization of H1-Lro1 to the ER, by removing a peptide from the Heh1 sequence, which is required for INM targeting of Heh1 (Meinema et al., 2011) (Figure 5B, panel 3) or (2) increasing DG levels at the nuclear membrane by overexpressing the active form of the PA phosphatase Pah1 or deleting the DG kinase *DGK1* increase TG levels and lead to the appearance of perinuclear LDs (Figures 5E and S3D, panel 3). Collectively, these data are consistent with a model where the INM can support TG synthesis during the PDS phase, when DG concentrates at this subdomain of the ER.

Nuclear Lro1-Derived TG Is Packed into LDs Associated Mostly with the Outer Nuclear Membrane

If TG, which is generated by Lro1 at the INM, can sustain cell viability during starvation, then this storage lipid has to become available to cytoplasmic organelles where its fatty acids are metabolized. To investigate this, we determined the spatial positioning of LDs with respect to the nuclear membrane in 4Δ H1-Lro1 cells during the PDS phase by using enhanced resolution Airyscan microscopy. As shown in Figure 5F, LDs were detected in the nucleoplasm in close proximity to the nuclear envelope in sequential z-slices that encompass the diameter of the LD. INM-associated LDs were also detected in 4Δ cells expressing wild-type Lro1 at the PDS phase (Figure S3E). In both strains, however, LDs associated with the INM were rare. We found that the majority of LDs in the H1-Lro1 strain associate with the outer nuclear membrane. Electron microscopy using a cryo-sectioning procedure of chemically fixed cells for the morphological examination of 4Δ H1-Lro1 cells showed that LDs were associated with the outer side of the nuclear envelope but failed to resolve the nature of the LD-nuclear membrane association (Figure 5G). High-pressure freezing electron microscopy confirmed that LDs in this strain associate with the outer nuclear membrane (Figure 5H). Taken together, our data are consistent with a model where TG is exported from the INM and accumulates in the ONM, where it is packed into mature LDs.

Compartmentalization of INM TG Synthesis Is Important for Maintenance of Nuclear Integrity

Our data show that Lro1 can support TG synthesis when it localizes throughout the entire INM (H1-Lro1). In wild-type cells, however, Lro1 activity is restricted to a specific subdomain of the INM. We therefore wondered why yeast cells maintained this localized targeting of Lro1. We hypothesized that, when not confined, LD formation at the nuclear envelope may disrupt nuclear morphology and other nuclear functions, in particular under conditions of enhanced TG synthesis. To test this hypothesis, we first confirmed that the different localization of the Lro1 mutants correlates with a distinct subcellular distribution of LDs in 4Δ cells. Indeed, consistent with its constitutive perinuclear localization, H1-Lro1-derived LDs appeared nearly exclusively associated with the nuclear envelope in the PDS phase or after supplementation of oleate. In contrast, under the same conditions, cells expressing 3xMBP-Lro1 displayed an increased number of cortical LDs (Figures 6A and 6B). Next, we evaluated nuclear morphology in these cells by measuring the size and shape of their nuclei. The average cross-sectional surface of nuclei from the 4Δ H1-Lro1 cells (1.93 ± 0.78 μm^2) showed a modest but significant decrease when compared with cells expressing the wild-type enzyme (2.08 ± 0.71 μm^2), while the opposite result was observed for 4Δ 3xMBP-Lro1 cells (2.19 ± 0.84 μm^2) (Figure 6C). To evaluate the nuclear shape, we measured nuclear circularity during the PDS phase. Circularity was significantly lower in 4Δ H1-Lro1 than in 4Δ Lro1 cells (Figure 6D). The nuclei of 4Δ 3xMBP-Lro1 cells displayed higher circularity than those of 4Δ H1-Lro1 cells although they displayed a modest decrease when compared to 4Δ Lro1 nuclei (Figure 6D). Thus, shifting Lro1 from the cortical ER to the INM decreases nuclear surface, consistent with a role of PDAT activity in organelle remodeling.

Next, we asked whether constitutive LD formation at the INM compromises nuclear homeostasis. Maintenance of nuclear envelope integrity and repair is mediated by the ESCRT-III machinery (Webster et al., 2014; Olmos et al., 2015; Vietri et al., 2015). We therefore examined whether ESCRT-III would be required for cell viability when the only source of TG synthesis, during fatty acid overload, would be at the INM. To test this, we deleted *VPS4*, a key component of ESCRT-III, in 4Δ H1-Lro1 and challenged the cells with oleate. While the single mutants of either *vps4*Δ or 4Δ H1-Lro1 grew in the presence of oleate, the double mutant 4Δ*vps4*Δ H1-Lro1 displayed a strong growth inhibition (Figure 6E). In contrast, 4Δ*vps4*Δ 3xMBP-LRO1 showed no defect, consistent with the notion that loss of viability is specifically linked to LD production at the INM. Since ESCRT-III is involved in membrane remodeling events in multiple organelles, we deleted *CHM7*, which is involved in the specific nuclear envelope recruitment of ESCRT-III (Webster et al., 2016; Gu et al., 2017). Consistently, the double mutant 4Δ*chm7*Δ H1-Lro1 displayed a growth defect in the presence of oleate when compared to 4Δ H1-Lro1 (Figure 6F). 4Δ H1-Lro1 displayed also a growth defect when lacking *NUP188*, a component of the inner ring of the nuclear pore complex, further supporting the requirement of a functional nuclear envelope during INM-derived LD formation. Collectively, these data suggest that biosynthetic production of TG is likely to cause stress to the INM. By restricting PDAT activity to the nucleolar membrane, cells maintain nuclear integrity.

DISCUSSION

Eukaryotic cells possess efficient mechanisms for TG synthesis and packing at the cytoplasmic ER. Depending on the cellular metabolic requirements, fatty acids stored in TGs can be used for energy production and/or membrane biogenesis. In budding yeast, the tight association of the nucleolus with the nuclear periphery defines a membrane subdomain, which has been implicated in nuclear shape, nucleophagy, and rDNA anchoring to the nuclear envelope. We find that the yeast acyltransferase Lro1 is active at this subdomain, generating TGs from phospholipid-derived fatty acids, in response to cell cycle and nutrient signals.

Several reports have documented the presence of intranuclear LDs, but the origin of the TG composing them, as well as their roles, remain elusive (Layerenza et al., 2013; Uzbekov and Roingeard, 2013; Cartwright et al., 2015; Grippa et al., 2015; Wolinski et al., 2015; Ohsaki et al., 2016; Romanauska and Köhler, 2018). The DGAT Dga1 makes the majority of TG in yeast cells at the stationary phase (Oelkers et al., 2002; Sandager et al., 2002). Under these conditions, we showed that Lro1 associates with, and concentrates at, the INM subdomain. Thus, our data support the notion of a restricted activity of Lro1 at the nuclear envelope, which may explain its limited contribution in bulk TG synthesis in wild-type cells during starvation (Oelkers et al., 2002). Given its topology, however, we cannot exclude that Dga1 is also contributing to nuclear TG synthesis. In fact, DGAT2 association with intranuclear LDs was proposed to mediate their expansion in hepatocyte-derived cell lines (Ohsaki et al., 2016).

Although it is generally accepted that TG storage in LDs takes place at the ER, our data reveal that cells can survive during

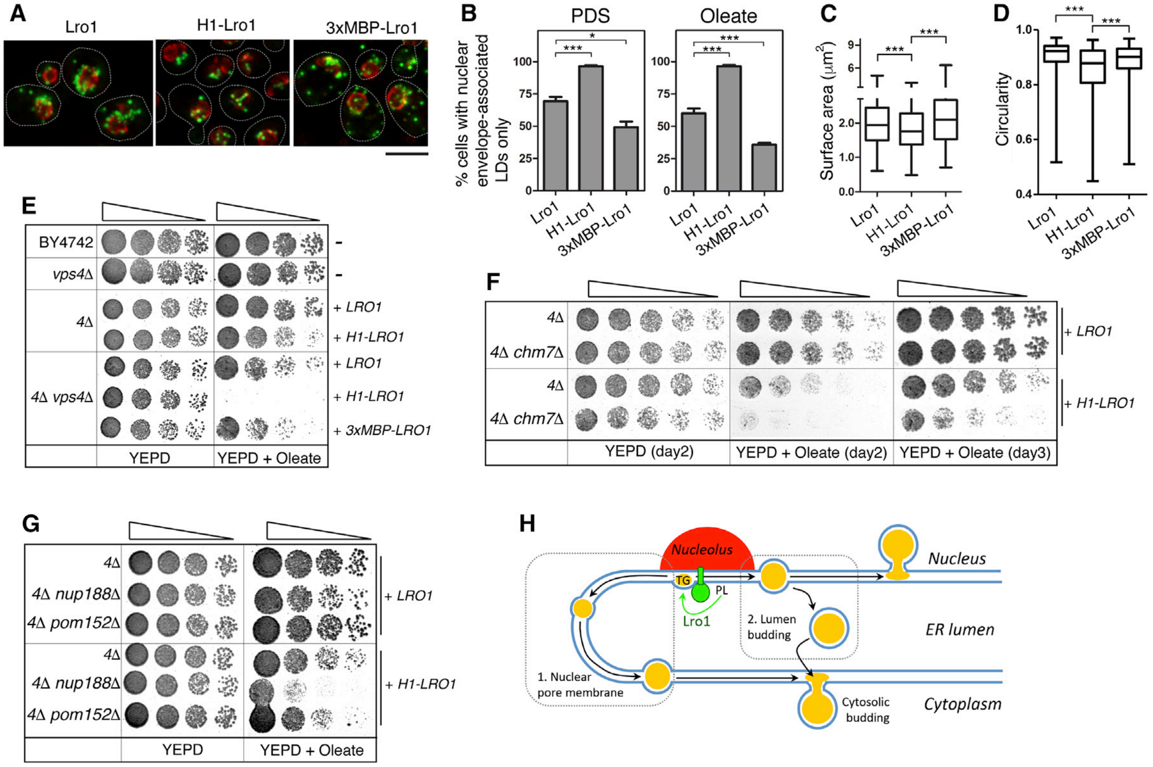

Figure 6. Compartmentalization of INM TG Synthesis Is Required to Maintain Nuclear Homeostasis

(A) Distribution of LDs, labeled by BODIPY 493/503, in 4Δ cells expressing Nup84-mCherry and the indicated Lro1 proteins during the PDS phase; the cell outlines are shown. Scale bar, 5 μm.

(B) Quantification of the association of LDs with the nuclear envelope in the strains shown in (A) (PDS phase; four experiments, n = at least 350 cells per strain) or in the same strains grown in exponential phase and incubated with glucose-containing media with 0.1% oleate for 2 h (three experiments, n = at least 400 cells per strain); data are means ± SDs.

(C) Quantification of nuclear envelope surface area in 4Δ cells expressing the indicated Lro1 proteins at the PDS phase; data are means from five experiments (n = at least 400 per strain counted) ± SDs.

(D) Quantification of nuclear envelope circularity in the samples from (A); data are means from six experiments and at least 400 cells per strain.

(E) Loss of *VPS4* inhibits growth of 4Δ H1-Lro1 cells in the presence of oleate. The indicated strains expressing the Lro1 constructs shown were grown to the exponential phase in glucose-containing medium, spotted on YEPD plates in the absence or presence of 1 mM oleate and grown for 2 days.

(F) Loss of *CHM7* inhibits growth of 4Δ H1-Lro1 cells in the presence of oleate. The specified strains were grown as described above.

(G) Loss of *NUP188*, but not *POM152*, inhibits growth of 4Δ H1-Lro1 cells in the presence of oleate. The specified strains were grown as described above.

(H) Model for the export of Lro1-derived TG to the outer nuclear membrane; see discussion for details. *$p < 0.05$, ***$p < 0.001$.

starvation when engineered to depend exclusively on TG generated at the INM. Given that the final destination of the TG-stored fatty acids during starvation are peroxisomes, mitochondria, and the vacuole, this result is consistent with the presence of a pathway that delivers nuclear TG to its cytoplasmic destinations. How such a process could operate will be the subject of future studies, but some scenarios can be hypothesized. Low levels of TG can be accommodated between the leaflets of a phospholipid bilayer and could diffuse through the nuclear pore membrane to the outer membrane, where they would be packed into mature LDs. Alternatively, budding of LDs toward the luminal side could channel them toward the outer nuclear membrane (Figure 6H). Our data on the requirement of nuclear membrane and pore integrity under conditions of enhanced TG synthesis at the INM is consistent with both scenarios. However, LDs can bud toward the nucleoplasm in mutants that affect the phospholipid composition of the ER and that of the LD monolayer, (Cartwright et al., 2015; Grippa et al., 2015; Romanauska and Köhler, 2018). Therefore, membrane phospholipid composition may be critical in determining the directionality of LD budding.

In yeast cells, the nuclear membrane expands to allow anaphase to take place within an intact nucleus. The membrane associated with the nucleolus can also expand in response to excess phospholipid synthesis, resulting in alterations of nuclear shape (Campbell et al., 2006; Witkin et al., 2012). Although the precise mechanisms of this process remain to be determined, one possibility could be that Lro1-mediated generation of either lysophospholipids or re-esterified phospholipids with a distinct fatty-acyl composition modifies the biophysical properties of this membrane subdomain and its dynamics. Because Lro1 targeting to this subdomain is prevented under conditions that require nuclear expansion, it is tempting to speculate that Lro1 plays a role in the regulation of this process. The pool of DG that is required for TG synthesis by Lro1 (Figures 1B and S3D) could be provided by Pah1, which is known to target the nuclear envelope at the diauxic shift (Barbosa et al., 2015), but other

enzymes could act in concert with Lro1 as well. We cannot also exclude the possibility that Lro1 controls nuclear organization through additional mechanisms that are independent of its catalytic activity. The highly basic N-domain of Lro1 faces the nucleoplasm and its interaction with nucleic acid or protein components of the nucleolus could restrict the expansion of its membrane subdomain.

It is conceivable that Lro1-mediated membrane remodeling could also play additional roles at the INM. Changes in nuclear membrane dynamics may be required for the removal of nuclear material during starvation conditions via piecemeal microautophagy (Roberts et al., 2003) and receptor-mediated nucleophagy (Mochida et al., 2015; Mostofa et al., 2018). Both processes take place in proximity to the nucleolus. However, we found that at least one of these two processes, receptor-mediated nucleophagy, proceeds during glucose starvation in the absence of Lro1 (data not shown). Lro1-mediated membrane remodeling could be also linked to nucleolar functions. For example, rDNA transcription and ribosome biogenesis are energy-consuming processes acutely inhibited during starvation, and they lead to a decrease in nucleolar size (Neumüller et al., 2013). Since the rDNA is physically tethered to the INM in yeast (Mekhail et al., 2008) Lro1 could remodel this membrane subdomain to facilitate nucleolar reorganization during starvation. Future studies will be needed to fully elucidate the role of INM lipid composition in nucleolar functions.

Previous studies reported that PDATs are present in fungi, green algae and plants, and they have a topology similar to that of Lro1 (Ståhl et al., 2004; Pan et al., 2015). Our analysis identified PDATs from two additional taxonomic groups, the flagellates Euglenozoa and the fungal-like Oomycetes (Table S2). However, the nucleolar-associated membrane has been described so far only in fungi, raising the question of PDAT function in other taxonomic groups. Notably, the green algal *C. reinhardtii* PDAT was proposed to use chloroplast membrane lipids to synthesize TG during starvation (Yoon et al., 2012). Similarly, the *Arabidopsis thaliana* Lro1 ortholog PDAT1, which also generates TG using phospholipids as acyl donors (Ståhl et al., 2004), re-localizes from the ER to chloroplasts following starvation induced by light deprivation (data not shown). Therefore, we speculate that PDATs respond to the need of remodeling or turnover of membranes. Given that the requirements of different cell types during stress are often distinct, PDATs may have evolved to target diverse organelles. It will be interesting to define the signals that govern PDAT dynamics in different cell types and examine whether animal cells, which lack apparent PDAT orthologs, maintain the ability to remodel membranes by a combination of phospholipase and acyltransferase activities.

STAR★METHODS

Detailed methods are provided in the online version of this paper and include the following:

- KEY RESOURCES TABLE
- LEAD CONTACT AND MATERIALS AVAILABILITY
- EXPERIMENTAL MODELS AND SUBJECT DETAILS
  - Yeast Strains, Plasmids, Media and Growth Conditions
- METHOD DETAILS
  - Fluorescence Microscopy
  - Fluorescence Recovery after Photobleaching
  - Automated Yeast Library Manipulations and High-Throughput Microscopy Screen
  - Electron Microscopy
  - Yeast Lipid Profiling
  - Immunoblotting
  - Flow Cytometry
  - Bioinformatics
- QUANTIFICATION AND STATISTICAL ANALYSIS
- DATA AND CODE AVAILABILITY

SUPPLEMENTAL INFORMATION

Supplemental Information can be found online at https://doi.org/10.1016/j.devcel.2019.07.009.

ACKNOWLEDGMENTS

We thank Nicholas Ktistakis, Delphine Larrieu, Helena Santos-Rosa, and Alison Schuldt for comments on the manuscript; Andrew Peden and Liesbeth Veenhoff for reagents; Matthew Gratian and Mark Bowen for help with microscopy; and Reiner Schulte and Chiara Cossetti for help with FACS analysis. This work was supported by a Wellcome Trust Seed award (108042) to S.S.; a Wellcome Trust Strategic award (100140) and an equipment grant (093026) to the Cambridge Institute for Medical Research; a Wellcome Trust Senior fellowship to D.B.S. (WT 107064); a Marie Skłodowska-Curie Cofund (713660), a Marie Skłodowska-Curie ITN (765912), a ALW Open Program (ALWOP.310), and ZonMW VICI grant (016.130.606) to F.R.; a ALW Open Program grant (ALWOP.355) to M.M.; a BBSRC grant (BB/M027252/1) to A.K.; a Volkswagen "Life" grant to M.S.; and a fellowship from the Gatsby Foundation (GAT3273/GLB) to P.A.W. J.R.E. was supported by Wellcome Trust (grant 086598). M.S. is an incumbent of the Dr. Gilbert Omenn and Martha Darling Professorial Chair in Molecular Genetics.

AUTHOR CONTRIBUTIONS

Conceptualization, S.S.; Experimental design, S.S., A.D.B., and P.A.W.; Yeast experimental work, A.D.B. and S.S.; Electron microscopy, M.M., J.R.E., and F.R.; FRAP and live microscopy, K.L. and D.B.S.; High-content imaging screen, M.S., L.G., and A.D.B.; Yeast lipid profiling, B.J.J. and A.K.; Bioinformatics, P.S.; Data analysis, S.S. and A.D.B. with the help of the other authors; Writing, S.S. and A.D.B.; Review/editing, all authors.

DECLARATION OF INTERESTS

The authors declare no competing interests.

Received: August 2, 2018
Revised: April 22, 2019
Accepted: July 3, 2019
Published: August 15, 2019

SUPPORTING CITATIONS

The following reference appear in the Supplemental Information: Albert et al., 2016.

REFERENCES

Albert, B., Knight, B., Merwin, J., Martin, V., Ottoz, D., Gloor, Y., Bruzzone, M.J., Rudner, A., and Shore, D. (2016). A molecular titration system coordinates ribosomal protein gene transcription with ribosomal RNA synthesis. Mol. Cell *64*, 720–733.

Barbosa, A.D., Sembongi, H., Su, W.M., Abreu, S., Reggiori, F., Carman, G.M., and Siniossoglou, S. (2015). Lipid partitioning at the nuclear envelope controls membrane biogenesis. Mol. Biol. Cell *26*, 3641–3657.

Barbosa, A.D., and Siniossoglou, S. (2017). Function of lipid droplet-organelle interactions in lipid homeostasis. Biochim. Biophys. Acta *1864*, 1459–1468.

Breslow, D.K., Cameron, D.M., Collins, S.R., Schuldiner, M., Stewart-Ornstein, J., Newman, H.W., Braun, S., Madhani, H.D., Krogan, N.J., and Weissman, J.S. (2008). A comprehensive strategy enabling high-resolution functional analysis of the yeast genome. Nat. Methods *5*, 711–718.

Campbell, J.L., Lorenz, A., Witkin, K.L., Hays, T., Loidl, J., and Cohen-Fix, O. (2006). Yeast nuclear envelope subdomains with distinct abilities to resist membrane expansion. Mol. Biol. Cell *17*, 1768–1778.

Cartwright, B.R., Binns, D.D., Hilton, C.L., Han, S., Gao, Q., and Goodman, J.M. (2015). Seipin performs dissectible functions in promoting lipid droplet biogenesis and regulating droplet morphology. Mol. Biol. Cell *26*, 726–739.

Choudhary, V., Jacquier, N., and Schneiter, R. (2011). The topology of the triacylglycerol synthesizing enzyme Lro1 indicates that neutral lipids can be produced within the luminal compartment of the endoplasmic reticulum: implications for the biogenesis of lipid droplets. Commun. Integr. Biol. *4*, 781–784.

Cohen, Y., and Schuldiner, M. (2011). Advanced methods for high-throughput microscopy screening of genetically modified yeast libraries. Methods Mol. Biol. *781*, 127–159.

Dahlqvist, A., Stahl, U., Lenman, M., Banas, A., Lee, M., Sandager, L., Ronne, H., and Stymne, S. (2000). Phospholipid : diacylglycerol acyltransferase: an enzyme that catalyzes the acyl-CoA-independent formation of triacylglycerol in yeast and plants. Proc. Natl. Acad. Sci. USA *97*, 6487–6492.

Dupont, N., Chauhan, S., Arko-Mensah, J., Castillo, E.F., Masedunskas, A., Weigert, R., Robenek, H., Proikas-Cezanne, T., and Deretic, V. (2014). Neutral lipid stores and lipase PNPLA5 contribute to autophagosome biogenesis. Curr. Biol. *24*, 609–620.

Finn, R.D., Clements, J., Arndt, W., Miller, B.L., Wheeler, T.J., Schreiber, F., Bateman, A., and Eddy, S.R. (2015). HMMER web server: 2015 update. Nucleic Acids Res. *43*, W30–W38.

Folch, J., Lees, M., and Sloane Stanley, G.H. (1957). A simple method for the isolation and purification of total lipides from animal tissues. J. Biol. Chem. *226*, 497–509.

Foresti, O., Rodriguez-Vaello, V., Funaya, C., and Carvalho, P. (2014). Quality control of inner nuclear membrane proteins by the Asi complex. Science *346*, 751–755.

Giaever, G., Chu, A.M., Ni, L., Connelly, C., Riles, L., Véronneau, S., Dow, S., Lucau-Danila, A., Anderson, K., André, B., et al. (2002). Functional profiling of the Saccharomyces cerevisiae genome. Nature *418*, 387–391.

Goodstein, D.M., Shu, S., Howson, R., Neupane, R., Hayes, R.D., Fazo, J., Mitros, T., Dirks, W., Hellsten, U., Putnam, N., et al. (2012). Phytozome: a comparative platform for green plant genomics. Nucleic Acids Res. *40*, D1178–D1186.

Griffith, J., Mari, M., De Mazière, A., and Reggiori, F. (2008). A cryosectioning procedure for the ultrastructural analysis and the immunogold labelling of yeast Saccharomyces cerevisiae. Traffic *9*, 1060–1072.

Grippa, A., Buxó, L., Mora, G., Funaya, C., Idrissi, F.Z., Mancuso, F., Gomez, R., Muntanyà, J., Sabidó, E., and Carvalho, P. (2015). The seipin complex Fld1/Ldb16 stabilizes ER-lipid droplet contact sites. J. Cell Biol. *211*, 829–844.

Gu, M., LaJoie, D., Chen, O.S., von Appen, A., Ladinsky, M.S., Redd, M.J., Nikolova, L., Bjorkman, P.J., Sundquist, W.I., Ullman, K.S., et al. (2017). LEM2 recruits CHMP7 for ESCRT-mediated nuclear envelope closure in fission yeast and human cells. Proc. Natl. Acad. Sci. USA *114*, E2166–E2175.

Haruki, H., Nishikawa, J., and Laemmli, U.K. (2008). The anchor-away technique: rapid, conditional establishment of yeast mutant phenotypes. Mol. Cell *31*, 925–932.

Herms, A., Bosch, M., Reddy, B.J., Schieber, N.L., Fajardo, A., Rupérez, C., Fernández-Vidal, A., Ferguson, C., Rentero, C., Tebar, F., et al. (2015). AMPK activation promotes lipid droplet dispersion on detyrosinated microtubules to increase mitochondrial fatty acid oxidation. Nat. Commun. *6*, 7176.

Hsu, T.H., Chen, R.H., Cheng, Y.H., and Wang, C.W. (2017). Lipid droplets are central organelles for meiosis II progression during yeast sporulation. Mol. Biol. Cell *28*, 440–451.

Huh, W.K., Falvo, J.V., Gerke, L.C., Carroll, A.S., Howson, R.W., Weissman, J.S., and O’Shea, E.K. (2003). Global analysis of protein localization in budding yeast. Nature *425*, 686–691.

Iwasa, S., Sato, N., Wang, C.W., Cheng, Y.H., Irokawa, H., Hwang, G.W., Naganuma, A., and Kuge, S. (2016). The phospholipid:diacylglycerol acyltransferase Lro1 is responsible for hepatitis C virus core-induced lipid droplet formation in a yeast model system. PLoS One *11*, e0159324.

Jacquier, N., Choudhary, V., Mari, M., Toulmay, A., Reggiori, F., and Schneiter, R. (2011). Lipid droplets are functionally connected to the endoplasmic reticulum in Saccharomyces cerevisiae. J. Cell Sci. *124*, 2424–2437.

Jain, S., Stanford, N., Bhagwat, N., Seiler, B., Costanzo, M., Boone, C., and Oelkers, P. (2007). Identification of a novel lysophospholipid acyltransferase in Saccharomyces cerevisiae. J. Biol. Chem. *282*, 30562–30569.

Janke, C., Magiera, M.M., Rathfelder, N., Taxis, C., Reber, S., Maekawa, H., Moreno-Borchart, A., Doenges, G., Schwob, E., Schiebel, E., et al. (2004). A versatile toolbox for PCR-based tagging of yeast genes: new fluorescent proteins, more markers and promoter substitution cassettes. Yeast *21*, 947–962.

Karanasios, E., Han, G.S., Xu, Z., Carman, G.M., and Siniossoglou, S. (2010). A phosphorylation-regulated amphipathic helix controls the membrane translocation and function of the yeast phosphatidate phosphatase. Proc. Natl. Acad. Sci. USA *107*, 17539–17544.

Khmelinskii, A., Blaszczak, E., Pantazopoulou, M., Fischer, B., Omnus, D.J., Le Dez, G., Brossard, A., Gunnarsson, A., Barry, J.D., Meurer, M., et al. (2014). Protein quality control at the inner nuclear membrane. Nature *516*, 410–413.

Koulman, A., Woffendin, G., Narayana, V.K., Welchman, H., Crone, C., and Volmer, D.A. (2009). High-resolution extracted ion chromatography, a new tool for metabolomics and lipidomics using a second-generation orbitrap mass spectrometer. Rapid Commun. Mass Spectrom. *23*, 1411–1418.

Kurat, C.F., Wolinski, H., Petschnigg, J., Kaluarachchi, S., Andrews, B., Natter, K., and Kohlwein, S.D. (2009). Cdk1/Cdc28-dependent activation of the major triacylglycerol lipase Tgl4 in yeast links lipolysis to cell-cycle progression. Mol. Cell *33*, 53–63.

Layerenza, J.P., González, P., García de Bravo, M.M., Polo, M.P., Sisti, M.S., and Ves-Losada, A. (2013). Nuclear lipid droplets: a novel nuclear domain. Biochim. Biophys. Acta *1831*, 327–340.

Longtine, M.S., McKenzie, A., 3rd, Demarini, D.J., Shah, N.G., Wach, A., Brachat, A., Philippsen, P., and Pringle, J.R. (1998). Additional modules for versatile and economical PCR-based gene deletion and modification in Saccharomyces cerevisiae. Yeast *14*, 953–961.

Lu, L., Koulman, A., Petry, C.J., Jenkins, B., Matthews, L., Hughes, I.A., Acerini, C.L., Ong, K.K., and Dunger, D.B. (2016). An unbiased lipidomics approach identifies early second trimester lipids predictive of maternal glycemic traits and gestational diabetes mellitus. Diabetes Care *39*, 2232–2239.

Meinema, A.C., Laba, J.K., Hapsari, R.A., Otten, R., Mulder, F.A., Kralt, A., van den Bogaart, G., Lusk, C.P., Poolman, B., and Veenhoff, L.M. (2011). Long unfolded linkers facilitate membrane protein import through the nuclear pore complex. Science *333*, 90–93.

Mekhail, K., Seebacher, J., Gygi, S.P., and Moazed, D. (2008). Role for perinuclear chromosome tethering in maintenance of genome stability. Nature *456*, 667–670.

Miles, S., Li, L., Davison, J., and Breeden, L.L. (2013). Xbp1 directs global repression of budding yeast transcription during the transition to quiescence and is important for the longevity and reversibility of the quiescent state. PLoS Genet. *9*, e1003854.

Mochida, K., Oikawa, Y., Kimura, Y., Kirisako, H., Hirano, H., Ohsumi, Y., and Nakatogawa, H. (2015). Receptor-mediated selective autophagy degrades the endoplasmic reticulum and the nucleus. Nature *522*, 359–362.

Mostofa, M.G., Rahman, M.A., Koike, N., Yeasmin, A.M., Islam, N., Waliullah, T.M., Hosoyamada, S., Shimobayashi, M., Kobayashi, T., Hall, M.N., et al.

(2018). CLIP and cohibin separate rDNA from nucleolar proteins destined for degradation by nucleophagy. J. Cell Biol. *217*, 2675–2690.

Neumüller, R.A., Gross, T., Samsonova, A.A., Vinayagam, A., Buckner, M., Founk, K., Hu, Y., Sharifpoor, S., Rosebrock, A.P., Andrews, B., et al. (2013). Conserved regulators of nucleolar size revealed by global phenotypic analyses. Sci. Signal. *6*, ra70.

Oelkers, P., Cromley, D., Padamsee, M., Billheimer, J.T., and Sturley, S.L. (2002). The DGA1 gene determines a second triglyceride synthetic pathway in yeast. J. Biol. Chem. *277*, 8877–8881.

Oelkers, P., Tinkelenberg, A., Erdeniz, N., Cromley, D., Billheimer, J.T., and Sturley, S.L. (2000). A lecithin cholesterol acyltransferase-like gene mediates diacylglycerol esterification in yeast. J. Biol. Chem. *275*, 15609–15612.

O'Hara, L., Han, G.S., Peak-Chew, S., Grimsey, N., Carman, G.M., and Siniossoglou, S. (2006). Control of phospholipid synthesis by phosphorylation of the yeast lipin Pah1p/Smp2p Mg2+-dependent phosphatidate phosphatase. J. Biol. Chem. *281*, 34537–34548.

Ohsaki, Y., Kawai, T., Yoshikawa, Y., Cheng, J., Jokitalo, E., and Fujimoto, T. (2016). PML isoform II plays a critical role in nuclear lipid droplet formation. J. Cell Biol. *212*, 29–38.

Olmos, Y., Hodgson, L., Mantell, J., Verkade, P., and Carlton, J.G. (2015). ESCRT-III controls nuclear envelope reformation. Nature *522*, 236–239.

Pan, X., Peng, F.Y., and Weselake, R.J. (2015). Genome-wide analysis of PHOSPHOLIPID: DIACYLGLYCEROL ACYLTRANSFERASE (PDAT) genes in plants reveals the eudicot-wide PDAT gene expansion and altered selective pressures acting on the core eudicot PDAT paralogs. Plant Physiol. *167*, 887–904.

Petschnigg, J., Wolinski, H., Kolb, D., Zellnig, G., Kurat, C.F., Natter, K., and Kohlwein, S.D. (2009). Good fat, essential cellular requirements for triacylglycerol synthesis to maintain membrane homeostasis in yeast. J. Biol. Chem. *284*, 30981–30993.

Popken, P., Ghavami, A., Onck, P.R., Poolman, B., and Veenhoff, L.M. (2015). Size-dependent leak of soluble and membrane proteins through the yeast nuclear pore complex. Mol. Biol. Cell *26*, 1386–1394.

Rambold, A.S., Cohen, S., and Lippincott-Schwartz, J. (2015). Fatty acid trafficking in starved cells: regulation by lipid droplet lipolysis, autophagy, and mitochondrial fusion dynamics. Dev. Cell *32*, 678–692.

Ren, J., Pei-Chen Lin, C., Pathak, M.C., Temple, B.R., Nile, A.H., Mousley, C.J., Duncan, M.C., Eckert, D.M., Leiker, T.J., Ivanova, P.T., et al. (2014). A phosphatidylinositol transfer protein integrates phosphoinositide signaling with lipid droplet metabolism to regulate a developmental program of nutrient stress-induced membrane biogenesis. Mol. Biol. Cell *25*, 712–727.

Riekhof, W.R., Wu, J., Jones, J.L., and Voelker, D.R. (2007). Identification and characterization of the major lysophosphatidylethanolamine acyltransferase in Saccharomyces cerevisiae. J. Biol. Chem. *282*, 28344–28352.

Roberts, P., Moshitch-Moshkovitz, S., Kvam, E., O'Toole, E., Winey, M., and Goldfarb, D.S. (2003). Piecemeal microautophagy of nucleus in Saccharomyces cerevisiae. Mol. Biol. Cell *14*, 129–141.

Romanauska, A., and Köhler, A. (2018). The inner nuclear membrane is a metabolically active territory that generates nuclear lipid droplets. Cell.

Ruggles, K.V., Turkish, A., Sturley, S.L., and Turkish. (2013). Making, baking, and breaking: the synthesis, storage, and hydrolysis of neutral lipids. Annu. Rev. Nutr. *33*, 413–451.

Sandager, L., Gustavsson, M.H., Ståhl, U., Dahlqvist, A., Wiberg, E., Banas, A., Lenman, M., Ronne, H., and Stymne, S. (2002). Storage lipid synthesis is non-essential in yeast. J. Biol. Chem. *277*, 6478–6482.

Shpilka, T., Welter, E., Borovsky, N., Amar, N., Mari, M., Reggiori, F., and Elazar, Z. (2015). Lipid droplets and their component triglycerides and steryl esters regulate autophagosome biogenesis. EMBO J. *34*, 2117–2131.

Ståhl, U., Carlsson, A.S., Lenman, M., Dahlqvist, A., Huang, B., Banas, W., Banas, A., and Stymne, S. (2004). Cloning and functional characterization of a phospholipid:diacylglycerol acyltransferase from Arabidopsis. Plant Physiol. *135*, 1324–1335.

Stone, E.M., Heun, P., Laroche, T., Pillus, L., and Gasser, S.M. (2000). MAP kinase signaling induces nuclear reorganization in budding yeast. Curr. Biol. *10*, 373–382.

Taddei, A., and Gasser, S.M. (2012). Structure and function in the budding yeast nucleus. Genetics *192*, 107–129.

Tamaki, H., Shimada, A., Ito, Y., Ohya, M., Takase, J., Miyashita, M., Miyagawa, H., Nozaki, H., Nakayama, R., and Kumagai, H. (2007). LPT1 encodes a membrane-bound O-acyltransferase involved in the acylation of lysophospholipids in the yeast Saccharomyces cerevisiae. J. Biol. Chem. *282*, 34288–34298.

Ungricht, R., and Kutay, U. (2017). Mechanisms and functions of nuclear envelope remodelling. Nat. Rev. Mol. Cell Biol. *18*, 229–245.

Uzbekov, R., and Roingeard, P. (2013). Nuclear lipid droplets identified by electron microscopy of serial sections. BMC Res. Notes *6*, 386.

Vietri, M., Schink, K.O., Campsteijn, C., Wegner, C.S., Schultz, S.W., Christ, L., Thoresen, S.B., Brech, A., Raiborg, C., and Stenmark, H. (2015). Spastin and ESCRT-III coordinate mitotic spindle disassembly and nuclear envelope sealing. Nature *522*, 231–235.

Walter, P., and Ron, D. (2011). The unfolded protein response: from stress pathway to homeostatic regulation. Science *334*, 1081–1086.

Wang, C.W., and Lee, S.C. (2012). The ubiquitin-like (UBX)-domain-containing protein Ubx2/Ubxd8 regulates lipid droplet homeostasis. J Cell Sci *125*, 2930–2939.

Wang, H., Airola, M.V., and Reue, K. (2017). How lipid droplets "TAG" along: glycerolipid synthetic enzymes and lipid storage. Biochim. Biophys. Acta *1862*, 1131–1145.

Webster, B.M., Colombi, P., Jäger, J., and Lusk, C.P. (2014). Surveillance of nuclear pore complex assembly by ESCRT-III/Vps4. Cell *159*, 388–401.

Webster, B.M., Thaller, D.J., Jäger, J., Ochmann, S.E., Borah, S., and Lusk, C.P. (2016). Chm7 and Heh1 collaborate to link nuclear pore complex quality control with nuclear envelope sealing. EMBO J. *35*, 2447–2467.

Witkin, K.L., Chong, Y., Shao, S., Webster, M.T., Lahiri, S., Walters, A.D., Lee, B., Koh, J.L., Prinz, W.A., Andrews, B.J., et al. (2012). The budding yeast nuclear envelope adjacent to the nucleolus serves as a membrane sink during mitotic delay. Curr. Biol. *22*, 1128–1133.

Wolinski, H., Hofbauer, H.F., Hellauer, K., Cristobal-Sarramian, A., Kolb, D., Radulovic, M., Knittelfelder, O.L., Rechberger, G.N., and Kohlwein, S.D. (2015). Seipin is involved in the regulation of phosphatidic acid metabolism at a subdomain of the nuclear envelope in yeast. Biochim. Biophys. Acta *1851*, 1450–1464.

Yoon, K., Han, D., Li, Y., Sommerfeld, M., and Hu, Q. (2012). Phospholipid:diacylglycerol acyltransferase is a multifunctional enzyme involved in membrane lipid turnover and degradation while synthesizing triacylglycerol in the unicellular green microalga Chlamydomonas reinhardtii. Plant Cell *24*, 3708–3724.

Zhang, D., and Oliferenko, S. (2013). Remodeling the nuclear membrane during closed mitosis. Curr. Opin. Cell Biol. *25*, 142–148.

STAR★METHODS

KEY RESOURCES TABLE

REAGENT or RESOURCE	SOURCE	IDENTIFIER
Antibodies		
Rabbit Polyclonal anti-GFP	A. Peden	N/A
Mouse monoclonal anti-HA	Abcam	Cat#Ab16918; RRID: AB_302562
Goat polyclonal horseradish Peroxidase (HRP)-conjugated anti-rabbit Immunoglobulin-specific	BD Biosciences	Cat#554021; RRID: AB_395213
Chemicals		
Rapamycin	LC Laboratories	Cat#R-5000
BODIPY 493/503	Thermo Fisher Scientific	Cat#D-3922
Nocodazole	Sigma	Cat#M1404
Oleic acid	Sigma	Cat#05508-5ML-F
α1-Mating Factor acetate salt	Sigma	Cat#T6901
CE_(18:0$_{d6}$)	QMX	Cat#D-5823
Ceramide_C16$_{d31}$	AVANTI	Cat#868516P
FA_C15:0$_{d29}$	QMX	Cat#D-4020
FA_C17:0$_{d33}$	QMX	Cat#D-5261
FA_C20:0$_{d39}$	QMX	Cat#D-1617
LPC_(C14:0$_{d42}$)	QMX	Cat#D-5885
PA_(C16:0$_{d31}$ / C18:1)	AVANTI	Cat#860453P
PC_(C16:0$_{d31}$ / C18:1)	AVANTI	Cat#860399P
PE_(C16:0$_{d31}$ / C18:1)	AVANTI	Cat#860374P
PG_(C16:0$_{d31}$ / C18:1)	AVANTI	Cat#860384P
PI_(C16:0$_{d31}$ / C18:1)	AVANTI	Cat#860042P
PS_(C16:0$_{d62}$)	AVANTI	Cat#860401P
SM_(C16:0$_{d31}$)	AVANTI	Cat#868584P
TG_(45:0$_{d29}$)	QMX	Cat#D-5265
TG_(48:0$_{d31}$)	QMX	Cat#D-5213
TG_(54:0$_{d35}$)	QMX	Cat#D-5217
Experimental Models: Organisms/Strains		
S. cerevisiae: BY4741 *MATa his3Δ0 leu2Δ0 met15Δ0 ura3Δ0*	Open Biosystems	BY4741
S. cerevisiae: BY4742 *MATαhis3Δ1 leu2Δ0 lys2Δ0 ura3Δ0*	Open Biosystems	BY4742
S. cerevisiae: BY4741 *lro1::KanMX*	This paper	SS2543
S. cerevisiae: BY4741 *lro1::KanMX NOP10-mCherry::HisMX6*	This paper	SS2754
S. cerevisiae: BY4741 *lro1::KanMX hrd1::hphNT1*	This paper	SS2825
S. cerevisiae: BY4741 *HIS3::pRS403-NOP1-RFP*	This paper	SS2907
S. cerevisiae: BY4741 *ale1::KanMX*	Open Biosystems	*ale1Δ*
S. cerevisiae: BY4741 *plb1::hphNT1 plb2::KanMX plb3::NatMX6 nte1::URA3 lro1::HIS3*	This paper	SS2410
S. cerevisiae: BY4742 *asi3::KanMX*	Open Biosystems	*asi3Δ*
S. cerevisiae: BY4742 *ctf19::KanMX*	Open Biosystems	*ctf19Δ*
S. cerevisiae: BY4742 *mcm21::KanMX*	Open Biosystems	*mcm21Δ*
S. cerevisiae: BY4741 *rad52::KanMX HIS3::pRS403-NOP1-RFP*	This paper	SS3031
S. cerevisiae: BY4742 *vps4::KanMX*	Open Biosystems	*vps4Δ*
S. cerevisiae: BY4741 *ALE1-GFP::hphNT1*	This paper	SS2874

(*Continued on next page*)

Continued

REAGENT or RESOURCE	SOURCE	IDENTIFIER
S. cerevisiae: BY4741 *LPL1-GFP::HIS3MX*	Huh et al., 2003	*LPL1-GFP*
S. cerevisiae: BY4741 *SLC1-GFP::HIS3MX*	Huh et al., 2003	*SLC1-GFP*
S. cerevisiae: *MATα his3Δ1 leu2Δ0 met15Δ0 ura3Δ0 lys2+/lys+ can1Δ::STE2pr-sp HIS5 lyp1Δ::STE3pr-LEU2 LRO1-GFP-NatMX6*	This paper	SS2654
S. cerevisiae: *MATα his3Δ1 leu2Δ0 lys2Δ0 ura3Δ0 met15Δ0 are1::KanMX are2:KanMX trp1::URA lro1::TRP dga1::Lox-HIS-Lox*	Jacquier et al., 2011	RSY3077 (a.k.a. 4Δ)
S. cerevisiae: 4Δ *dgk1::HIS3MX6*	Barbosa et al., 2015	SS2468
S. cerevisiae: 4Δ *chm7::NatMX6*	This paper	SS2951
S. cerevisiae: 4Δ *vps4::NatMX6*	This paper	SS2953
S. cerevisiae: 4Δ *nup188::NatMX6*	This paper	SS2966
S. cerevisiae: 4Δ *pom152::NatMX6*	This paper	SS2964
S. cerevisiae: 4Δ *FAA4-GFP-HISMX6*	This paper	SS2922
S. cerevisiae: W303 *MATα tor1-1 fpr1::NAT*	EUROSCARF	K14708
S. cerevisiae: W303 *MATα tor1-1 fpr1::NAT HEH1-2xFKBP12::TRP1 lro1::KanMX*	This paper	SS2745
S. cerevisiae: W303 *MATα tor1-1 fpr1::NAT HEH1-2xFKBP12::TRP1 lro1::KanMX dga1::hphNT1*	This paper	SS2991
S. cerevisiae: W303 *MATα tor1-1 fpr1::NAT HEH1-2xFKBP12::TRP1 lro1::KanMX dgk1::HIS3*	This paper	SS3037
S. cerevisiae: W303 *MATα tor1-1 fpr1::NAT RPL13A-2×FKBP12::TRP1*	EUROSCARF	HHY168
S. cerevisiae: W303 *MATα tor1-1 fpr1::NAT RPL13A-2×FKBP12::TRP1 RPA135-FRB::KanMX*	This paper	SS2837
Recombinant DNA		
LRO1 under control of *LRO1* promoter in *CEN/URA3* vector	This paper	YCplac33-*LRO1*
LRO1-GFP under control of *LRO1* promoter in *CEN/URA3* vector	This paper	YCplac33-*LRO1-GFP*
LRO1-mCherry under control of *LRO1* promoter in *CEN/URA3* vector	This paper	YCplac33-*LRO1-mCherry*
LRO1-6xHA under control of *NOP1* promoter in *CEN/URA3* vector	This paper	YCplac33-*NOP1*pr-*LRO1-6xHA*
LRO1-GFP under control of *NOP1* promoter in *CEN/URA3* vector	This paper	YCplac33-*NOP1*pr-*LRO1-GFP*
4xIgGb-LRO1Δ[2-77]-GFP under control of *NOP1* promoter in *CEN/URA3* vector	This paper	YCplac33-*NOP1*pr-*4xIgGb-LRO1-GFP*
1xMBP-LRO1-GFP under control of *NOP1* promoter in *CEN/URA3* vector	This paper	YCplac33-*NOP1*pr-*1xMBP-LRO1-GFP*
2xMBP-LRO1-GFP under control of *NOP1* promoter in *CEN/URA3* vector	This paper	YCplac33-*NOP1*pr-*2xMBP-LRO1-GFP*
3xMBP-LRO1-GFP under control of *NOP1* promoter in *CEN/URA3* vector	This paper	YCplac33-*NOP1*pr-*3xMBP-LRO1-GFP*
LRO1[1-79]-GFP under control of *LRO1* promoter in *CEN/URA3* vector	This paper	YCplac33-*LRO1[1-79]-GFP*
LRO1[1-103]-GFP under control of *LRO1* promoter in *CEN/URA3* vector	This paper	YCplac33-*LRO1[1-103]-GFP*
LRO1[44-AAAA-47; 71-AAAWA-75]-GFP under control of *LRO1* promoter in *CEN/URA3* vector	This paper	YCplac33-*LRO1-NLS-GFP*
LRO1[1-79; 44-AAAA-47; 71-AAAWA-75]-GFP under control of *LRO1* promoter in *CEN/URA3* vector	This paper	YCplac33-*LRO1[1-79]-NLS-GFP*
LRO1[1-103; 44-AAAA-47; 71-AAAWA-75]-GFP under control of *LRO1* promoter in *CEN/URA3* vector	This paper	YCplac33-*LRO1[1-103]-NLS-GFP*

(*Continued on next page*)

Continued

REAGENT or RESOURCE	SOURCE	IDENTIFIER
FRB-LRO1-GFP under control of *NOP1* promoter in *CEN/URA3* vector	This paper	YCplac33-*NOP1*pr-*FRB-LRO1-GFP*
FRB-LRO1[S324A]-GFP under control of *NOP1* promoter in *CEN/URA3* vector	This paper	YCplac33-*NOP1*pr-*FRB-LRO1-S324A-GFP*
FRB-GFP under control of *NOP1* promoter in *CEN/LEU2* vector	This paper	YCplac111-*NOP1*pr-*FRB-GFP*
FRB-3xMBP-LRO1-GFP under control of *NOP1* promoter in *CEN/URA3* vector	This paper	YCplac33-*NOP1*pr- *FRB-3xMBP-LRO1-GFP*
LRO1[S324A]-GFP under control of *NOP1* promoter in *CEN/URA3* vector	This paper	YCplac33-*NOP1*pr-*LRO1-S324A-GFP*
LRO1 under control of *NOP1* promoter in *CEN/URA3* vector	This paper	YCplac33-*NOP1*pr-*LRO1*
HEH1[163-454]-LRO1Δ[2-77] under control of *NOP1* promoter in *CEN/URA3* vector	This paper	YCplac33-*NOP1*pr-*H1-LRO1*
HEH1[163-454]-LRO1Δ[2-77]-GFP under control of *NOP1* promoter in *CEN/URA3* vector	This paper	YCplac33-*NOP1*pr-*H1-LRO1-GFP*
HEH1[163-225]-LRO1Δ[2-77] under control of *NOP1* promoter in *CEN/URA3* vector	This paper	YCplac33-*NOP1*pr-*H1Δ[UP]-LRO1*
HEH1[163-225]-LRO1Δ[2-77]-GFP under control of *NOP1* promoter in *CEN/URA3* vector	This paper	YCplac33-*NOP1*pr-*H1Δ[UP]-LRO1-GFP*
3xMBP-LRO1 under control of *NOP1* promoter in *CEN/URA3* vector	This paper	YCplac33-*NOP1*pr-*3xMBP-LRO1*
LRO1 under control of *GAL1/10* promoter in 2μ/*LEU2* vector	This paper	YEplac181 *GAL1/10-LRO1*
LRO1[S324A] under control of *GAL1/10* promoter in 2μ/*LEU2* vector	This paper	YEplac181 *GAL1/10-LRO1-S324A*
PAH1-7A under control of *GAL1/10* promoter in 2μ/*LEU2* vector	O'Hara et al., 2006	YEplac181-*GAL1/10-PAH1-7A*
NTE1-GFP under control of *NTE1* promoter in *CEN/LEU2* vector	This paper	YCplac111-*NTE1-GFP*
SEC63-mCherry under control of *SEC63* promoter in *CEN/LEU2* vector	This paper	YCplac111-*SEC63-mCherry*
PUS1-GFP under control of *PUS1* promoter in *CEN/LEU2* vector	This paper	YCplac111-*PUS1-GFP*
NOP1-RFP under control of *NOP1* promoter in *CEN/TRP1* vector	This paper	pRS314-*NOP1-RFP*
NOP1-RFP under control of *NOP1* promoter in *CEN/HIS3* vector	This paper	pRS313-*NOP1-RFP*
NUP84-mCherry under control of *NUP84* promoter in *CEN/LEU2* vector	Barbosa et al., 2015	YCplac111-*NUP84-mCherry*

LEAD CONTACT AND MATERIALS AVAILABILITY

Further information and requests for resources and reagents should be directed to and will be fulfilled by the Lead Contact, Symeon Siniossoglou (ss560@cam.ac.uk).

EXPERIMENTAL MODELS AND SUBJECT DETAILS

Yeast Strains, Plasmids, Media and Growth Conditions

Unless otherwise specified, reagents were obtained from Sigma (St. Louis, MO). Yeast strains and plasmids are described in the Key Resources Table. Yeast plasmids were generated using standard PCR and cloning techniques. Cells were transformed using the lithium acetate method. Gene deletions and epitope tagging by chromosomal integration were generated by one-step polymerase chain reaction (PCR)-based method (Longtine et al., 1998; Janke et al., 2004; Haruki et al., 2008) and confirmed by PCR. In most experiments, cells were grown overnight at 30°C in synthetic complete (SC) medium containing 2% glucose, 0.2% yeast nitrogen base (YNB, Difco, BD, Franklin Lakes, NJ), 0.6% ammonium sulfate and amino acids drop-out (60 mg/L leucine, 55 mg/L adenine, 55 mg/L uracil, 55 mg/L tyrosine, 20 mg/L of arginine, 10 mg/L histidine, 60 mg/L isoleucine, 40 mg/L lysine, 60 mg/L phenylalanine, 50 mg/L threronine, 10 mg/L methionine, 40 mg/L tryptophan) to exponential phase (to OD_{600nm} 0.4-0.6), PDS phase (inoculated at

OD_{600nm} 0.05 and grown for approximately 15h to OD_{600nm} 4-6), or the indicated OD_{600nm}, from fresh pre-cultures, according to the previously determined growth rate in this medium (Barbosa et al., 2015). For stationary phase survival assays, cells were grown in minimal medium (MM) with 0.17% yeast nitrogen base (Difco, BD, Franklin Lakes, NJ), 0.5% ammonium sulfate and amino acids drop-out (20 mg/L uracil, 20 mg/L histidine, 20 mg/L methionine, 30 mg/L leucine, and 30 mg/L lysine) to PDS phase ($OD_{600nm} \sim 4$), and viability was measured every 24 hours by standard dilutions on YEP plates [2% glucose, 2% bactopeptone (BD, Franklin Lakes, NJ), 1% yeast extract (BD, Franklin Lakes, NJ)] with 2% glucose (YEPD medium) and 2% agar (Biogene, Kimbolton, UK). When required, SC and MM media lacked the appropriate amino acids for plasmid selection.

For *GAL1/10* promoter-mediated overexpression, cells were first pre-grown in selective SC medium with 2% glucose, grown overnight in selective SC medium with 2% raffinose, and transferred to selective SC medium with 2% galactose for the indicated times.

LD biogenesis in the presence of glucose was induced by growing the cells in YEPD with 1 mM oleic acid and 1% tergitol (solid media), or 0.1% oleic acid (3.2 mM oleate) and 0.2% Tween-80 pH 6.0 (in liquid media). Oleic acid uptake was confirmed by growth inhibition of the 4Δ strain (Petschnigg et al., 2009).

Growth assays on plates were performed using cells at the growth phase and media indicated in the figure legends. Serial dilutions were spotted onto the appropriate plates and incubated at 30°C for 2–4 days.

METHOD DETAILS

Fluorescence Microscopy

Lro1-GFP localization under stress conditions was tested transferring exponential cells for 1 h or 2 h into SC medium lacking a carbon source (carbon starvation), nitrogen starvation medium (2% glucose, 0.17% YNB), SC medium with 2% glycerol as carbon source (respiratory growth), or H_2O. An overnight culture grown to PDS phase was used as control, and targeting to the nucleolus was expressed as percentage of targeting in PDS. Enrichment of Lro1-GFP at the nucleolus in exponential phase was determined in asynchronous cultures. Rapamycin-induced heterodimerization of FKBP12 with the FRB domain of human mTOR was performed as previously described in the anchor-away technique (Haruki et al., 2008). Briefly, cells were grown in selective SC medium to exponential phase and treated with 1 μg/ml rapamycin for 30 min. In nocodazole-mediated cell cycle arrest experiments, *GAL1/10* promoter-mediated overexpression was induced as described above but growing the cells in YEP media with 2% galactose for 3 h before adding 15 μg/mL nocodazole for 2 h; in the anchor-away strains, cells were first transferred to YEPD media and treated with 1 μg/ml rapamycin for 30 min before adding 15 μg/mL nocodazole for 2 h. For alpha-factor cell cycle arrest, cells were treated with 10 μM alpha-factor for 2 h in YEPD. Lipid droplets were stained with 1.25 μg/ml BODIPY 493/503 for 10 min at room temperature.

Cells grown to the indicated growth phases were pelleted and immediately imaged live at room temperature in a Zeiss AxioImager.Z2 epifluorescence upright microscope with a 100× Plan-Apochromatic 1.4 numerical aperture (NA) objective lens (Carl Zeiss Ltd, Jena, Germany). Images were recorded using a large chip sCMOS mono camera for sensitive fluorescence imaging (ORCA Flash 4.0v2, Hamamatsu, Hamamatsu, Japan), saved by Zeiss ZEN2.3 software (Blue edition, Carl Zeiss Ltd, Jena, Germany) and exported to Adobe Photoshop (Adobe, San Jose, CA). Where indicated, cells were mounted on a 1% agarose pad and imaged using a Zeiss LSM880 confocal microscope with Airyscan and the ZEN2 software (Carl Zeiss Ltd, Jena, Germany). Cells were visualized from the periphery by taking 0.2 μm step slices and 50% overlap, and 0.187 μm axial resolution. All microscopy images were captured blindly and quantifications were performed on fields obtained from independent experiments.

For three-dimensional (3D) analysis, through-focus image series were deconvolved with Volocity 6.3 (PerkinElmer, Waltham, MA) using calculated point-spread functions and 3D iterative restoration processing to form 3D image stacks. Stacks were then visualized using Volocity 6.3 (PerkinElmer, Waltham, MA) to generate the 3D images or movies showing the organelles at different positions. For circularity and surface of the nucleus measurements, through-focus image series were deconvolved with Volocity 6.3 (PerkinElmer, Waltham, MA) and merged in 2D images showing the brightest intensity through z, which were exported and analysed in ImageJ 1.47v (NIH, Bethesda, MD).

Time-lapse imaging was performed in a Leica TCS SP8 confocal microscope. Yeast cells (4Δ) expressing Faa4-GFP, Nop1-RFP, and Lro1 under its endogenous promoter in a *CEN* vector, were grown in selective media to PDS phase and imaged on 2% agar pads. Images were acquired using a 63x oil immersion objective lens with an 8x zoom factor at 30 seconds intervals using white light laser at 488nm and 578nm for the excitation of GFP and RFP, respectively. Videos were generated using Imaris at a rate of three frames/second.

Fluorescence Recovery after Photobleaching

Photobleaching experiments were performed on a Leica TCS SP8 confocal microscope with the optional FRAP Booster enabled. Yeast *lro1Δ* cells expressing LRO1-GFP from *NOP1* promoter were pelleted and imaged at room temperature at early PDS phase. Images were acquired using a 63x oil immersion objective lens. After acquiring two images at 5-sec intervals, selected regions of interest were photobleached with 3 iterations of 100% laser power (white light laser) at 488 nm. The fluorescence intensity of GFP at the regions of interest was recorded for another 13 frames at 20 sec intervals. For data analyses, the fluorescence intensity of GFP was corrected by the percentage loss of GFP fluorescence intensity obtained from cells under identical conditions but without a photobleaching event. To calculate halftime of recovery ($T_{1/2}$), exponential FRAP curve fitting (non-linear regression, one way

association) was obtained using GraphPad Prism after excluding the two pre-bleaching measurement points. Mobile fraction of LRO1-GFP was calculated by subtracting the % of fluorescence intensity at T0 (I0) from the plateau level (Ie); while, immobile fraction was calculated by subtracting the mobile fraction from 100%.

Automated Yeast Library Manipulations and High-Throughput Microscopy Screen

Lro1-GFP, expressed from the *NOP1* promoter in a CEN/ARS *URA3* vector, was introduced in the KanMX deletion and DAmP collections (Giaever et al., 2002; Breslow et al., 2008) by Synthetic Genetic Array, by standard procedures previously described (Cohen and Schuldiner, 2011), using the RoToR bench-top colony arrayer (Singer Instruments, Roadwater, Watchet, UK). Cells were imaged, at room temperature at PDS phase in SC medium lacking uracil, using the automated inverted fluorescent microscopic ScanR system (Olympus, Waltham, Massachusetts, USA), with a 60× air lens, for GFP (excitation, 490/20 nm; emission, 535/50 nm) and brightfield channels. After acquisition, images were manually reviewed using the ImageJ software (NIH, Bethesda, MD). Cells showing increased Lro1-GFP signal in the ER, or overall decreased targeting to the nucleolus without enrichment at other subcellular localizations were selected for further analysis. The data obtained were analysed with GO (gene ontology) term finder of Saccharomyces Genome Database to determine GO term enrichment (https://www.yeastgenome.org/goTermFinder), and selecting the "Process" ontology aspect. Based on this analysis, Lro1-GFP localization was then manually inspected in mutants of interest (see Table S1).

Electron Microscopy

Cells were grown to the indicated growth phases, chemically fixed, embedded in 12% gelatin and cryo-sectioned as described previously (Griffith et al., 2008). Ultrathin cryo-sections were collected with a 1:1 mixture of 2% methylcellulose and 2.3 M, sucrose 120 mM PIPES, 50 mM HEPES, pH 6.9, 4 mM $MgCl_2$, 20 mM EGTA, and layered on Formvar/carbon coated 100 mesh copper grids. Immunological reactions were performed using a polyclonal anti-HA and a protein A-gold 10 nm conjugate (Cell Microscopy Center, Utrecht, The Netherlands). To determine protein localization, the distribution of gold particles was counted in 100 cells. The relative distribution of Lro1-6xHA to the cortical and the nuclear ER, was performed by counting the number of gold particles associated to these two cellular sub-compartments, on cell profiles randomly screened from immuno-labeled cell sections derived from at least three different grids. Gold particles were assigned to a compartment when no further than 10 nm away from its limiting membrane.

For high-pressure freezing electron microscopy, 4Δ H1-Lro1 cells were grown to PDS phase, pelleted and resuspended in a minimum volume of 20% BSA + 5% FBS/PBS to form a paste, and this paste was pipetted into flat specimen carriers and high-pressure frozen with a Leica EM PACT2. Freeze-substitution was performed with a Leica EM AFS2, and pellets were immersed in 0.1% tannic acid/acetone at -90°C for 24 hours, before being replaced by 2% osmium/ 0.1% UA (methanolic)/acetone for 48 hours at -90°C. Samples were then gradually warmed as follows; warmed to -56°C at a rate of 5°C/hour, held at -56°C for 12 hours, warmed to -20°C at a rate of 5°C/hour, held at -20°C for 12 hours, warmed to +4°C at a rate of 5°C/hour, held at +4°C for 4 hours. Pellets were washed three times with acetone before being gradually infiltrated with Spurr's resin over a period of 4 days. Ultrathin sections were cut using a diamond knife mounted to a Reichert Ultracut S ultramicrotome, and floating sections were collected onto copper grids. Grids were poststained first with both 2% uranyl acetate/70% methanol for 4 minutes and then with lead citrate for 4 minutes. Sections were viewed on an FEI Tecnai transmission electron microscope at a working voltage of 80 kV.

Yeast Lipid Profiling

Yeast cells were prepared for mass spectrometry analysis as previously described (Folch et al., 1957) with minor modifications. Briefly, 50 mg of yeast cells were re-suspended in 1 mL of 2:1 chloroform-methanol mixture (v/v), with the addition of 150 μL of the following internal standard solution (approximately 10 to 50 μM in methanol): CE_(18:0_{d6}), Ceramide_C16_{d31}, FA_C15:0_{d29}, FA_C17:0_{d33}, FA_C20:0_{d39}, LPC_(C14:0_{d42}), PA_(C16:0_{d31} / C18:1), PC_(C16:0_{d31} / C18:1), PE_(C16:0_{d31} / C18:1), PG_(C16:0_{d31} / C18:1), PI_(C16:0_{d31} / C18:1), PS_(C16:0_{d62}), SM_(C16:0_{d31}), TG_(45:0_{d29}), TG_(48:0_{d31}), and TG_(54:0_{d35}). The cells were homogenized with 100 mg 0.5 mm diameter glass beads (BioSpec Products, Bartlesville Oklahoma, USA) in a FastPrep-24 instrument (MP Biomedicals, Santa Ana California, USA), using five short pulses at 5 m/s for 1 min; with one minute on ice between each pulse to prevent over-heating. Then 400 μL of sterile water was added to the homogenates, vortexed for 1 min, and then centrifuged at 13,200 rpm for 10 minutes. The organic layer was collected in a 2 mL amber glass vial (Agilent Technologies, Santa Clara California, USA) and the remaining mixture was then treated with a second lipid extraction following the same procedure. The two organic layers were combined and air-dried overnight in a fume hood.

For lipid analysis, we used a method previously described with minor modification (Koulman et al., 2009; Lu et al., 2016). Full chromatographic separation of intact lipids was achieved using Shimadzu HPLC System (Shimadzu UK Limited, Milton Keynes, United Kingdom) with the injection of 10 μL onto an Acquity UPLC® CSH C18 column; 1.7 μm, I.D. 2.1 mm X 50 mm, maintained at 55°C. Mobile phase A was 6:4, acetonitrile and water with 10 mM ammonium formate. Mobile phase B was 9:1, 2-propanol and acetonitrile with 10 mM ammonium formate. The flow was maintained at 500 μL per minute through the following gradient: 0.00 minutes_40% mobile phase B; 0.40 minutes_43% mobile phase B; 0.45 minutes_50% mobile phase B; 2.40 minutes_54% mobile phase B; 2.45 minutes_70% mobile phase B; 7.00 minutes_99% mobile phase B; 8.00 minutes_99% mobile phase B; 8.3 minutes_40% mobile phase B; 10 minutes_40% mobile phase B. The sample injection needle was washed using 9:1, 2-propanol and acetonitrile with 0.1 % formic acid.

The mass spectrometer used was the Thermo Scientific Exactive Orbitrap with a heated electrospray ionisation source (Thermo Fisher Scientific, Hemel Hempstead, UK). The mass spectrometer was calibrated immediately before sample analysis using positive

and negative ionisation calibration solution (recommended by Thermo Scientific). Additionally, the heated electrospray ionisation source was optimised at 50:50 mobile phase A to mobile phase B for spray stability (capillary temperature; 380°C, source heater temperature; 420°C, sheath gas flow; 60 (arbitrary), auxiliary gas flow; 20 (arbitrary), sweep gas; 5 (arbitrary), source voltage; 3.5 kV. The mass spectrometer resolution was set to 25,000 with a full-scan range of m/z 150 to 1200 Da, with continuous switching between positive and negative mode.

Lipid quantification was achieved using the area under the curve (AUC) of the corresponding high resolution extracted ion chromatogram (with a window of ± 8 ppm) at the indicative retention time. The lipid analyte AUC relative to the internal standard AUC for that lipid class was used to semi-quantify and correct for any extraction/instrument variation. The normalized analyte intensities (analyte to internal standard ratios) were then expressed as a percentage of the total lipids extracted from that sample (mol %).

Immunoblotting

Yeast cells (approximately 12 OD_{600}) were pelleted, washed with sterile water, and lysed in 100 μl SDS-sample buffer with 0.5 mm diameter glass beads (BioSpec Products, Bartlesville, OK) by two rounds of boiling for 2 min and vortexing for 30 sec. Protein extracts were centrifuged at 13,000 rpm for 15 min, and the supernatants analysed by Western blot. Western blot signals were developed using ECL (GE Healthcare, Little Chalfont UK).

Flow Cytometry

Quantification of BODIPY by flow cytometry was performed as previously described (Barbosa et al., 2015). Briefly, cells were fixed for 30 min at room temperature with 3.7% formaldehyde, washed once with phosphate-buffered saline, and incubated with 10 μM BODIPY 493/503 for 10 min at room temperature. Labelling was immediately measured using the FL-1 detector of a FACSCalibur flow cytometer (BD Biosciences, San Jose, CA), and the results analysed with FlowJo software, version 9 (Tree Star, Ashland, OR).

Bioinformatics

For the identification of PDATs, the UniProt Knowledgebase (UniProtKB) was searched with the search term phospholipid:diacylglycerol acyltransferase at http://www.uniprot.org/. Partial sequences, sequences with zero or multiple transmembrane domains were removed. In order to extend the number of PDATs, we performed a phmmer search on the HMMER web server at https://www.ebi.ac.uk/Tools/hmmer/ (Finn et al., 2015) using the *S. cerevisiae* Lro1 (UniProtKB accession P40345) sequence to query UniProtKB. Additional plant PDATs, identified in Pan et al. 2015 were obtained from the Phytozome resource (Goodstein et al., 2012).

QUANTIFICATION AND STATISTICAL ANALYSIS

Unless otherwise stated, data were obtained from at least three independent repeats. The micrographs presented are representative of the results obtained. Signal intensity, surface and circularity were measured using ImageJ 1.47v (NIH, Bethesda, MD). Results were expressed as mean ± standard deviation. Data were analysed with unpaired, two-tailed t tests or one-way ANOVA with Tukey's multiple comparison test when more than two groups were compared using GraphPad Prism 5 software (GraphPad, La Jolla, CA). Statistical significance was defined as: *, $p < 0.05$; **, $p < 0.01$; and ***, $p < 0.001$.

DATA AND CODE AVAILABILITY

Datasets associated with this study are provided in Tables S1 and S2.

Developmental Cell, Volume *50*

Supplemental Information

Compartmentalized Synthesis of Triacylglycerol

at the Inner Nuclear Membrane

Regulates Nuclear Organization

Antonio D. Barbosa, Koini Lim, Muriel Mari, James R. Edgar, Lihi Gal, Peter Sterk, Benjamin J. Jenkins, Albert Koulman, David B. Savage, Maya Schuldiner, Fulvio Reggiori, Philip A. Wigge, and Symeon Siniossoglou

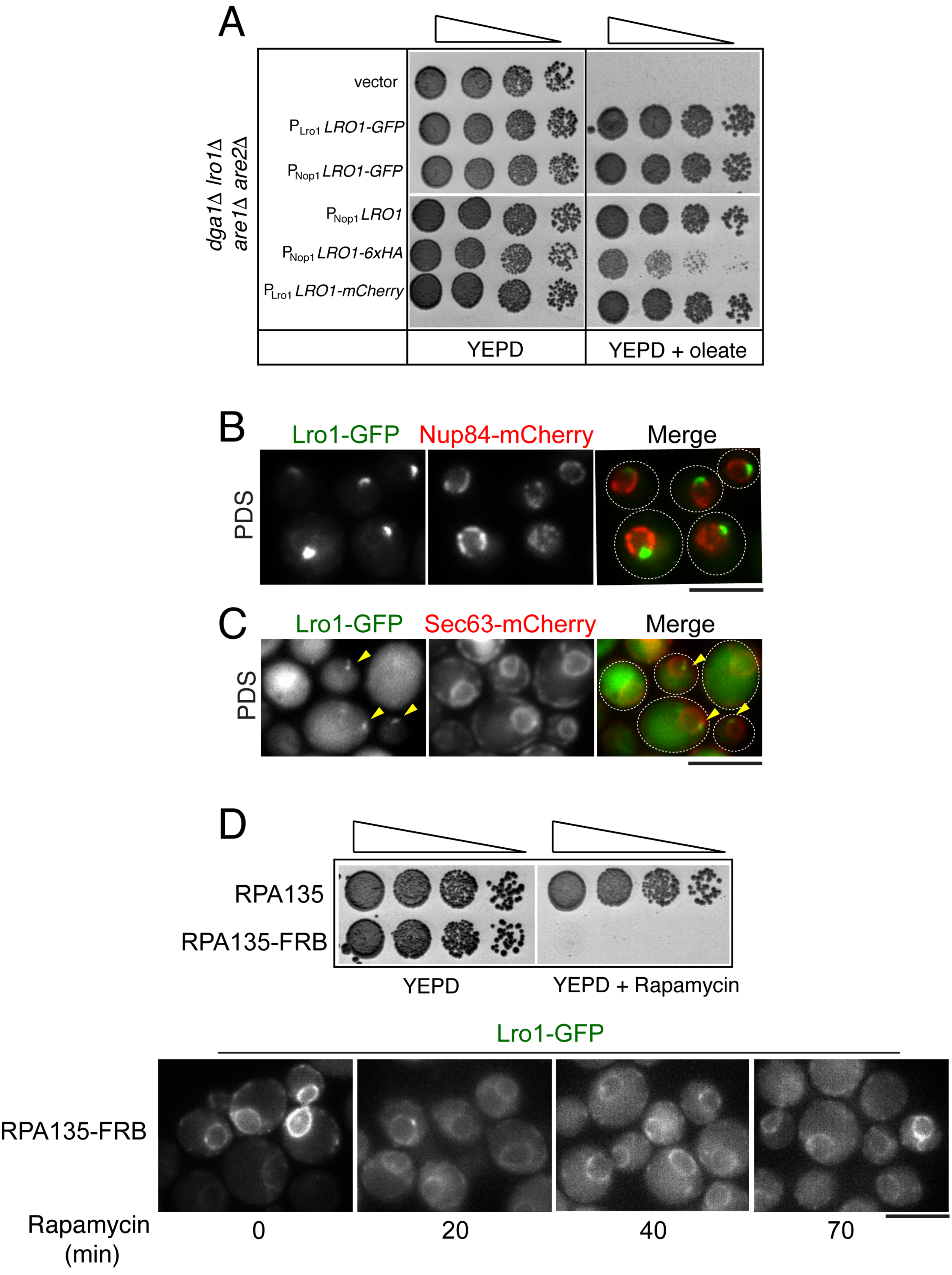

Figure S1. Related to Figure 1

Figure S1: Targeting of Lro1 to the nucleolar-associated membrane. (A) *dga1*Δ *lro1*Δ *are1*Δ *are2*Δ (4Δ) cells expressing the Lro1 epitope-tagged plasmids that were used in this study, were grown to the exponential phase and 0.2 $OD_{(600)}$ of cells were spotted on YEPD plates with or without oleate. 4Δ cells lack LDs and as a result lose viability when challenged with free fatty acid. Growth indicates a functional Lro1 fusion. P_{LRO1}; expression under the control of the *LRO1* promoter; P_{NOP1}; expression under the control of the *NOP1* promoter. (B) Co-localization of Lro1-GFP expressed under the control of the *NOP1* promoter, and the nuclear pore complex protein Nup84-mCherry in wild-type cells at the PDS phase. The outlines of cells are depicted. (C) Co-localization of Lro1-GFP integrated at its endogenous chromosomal locus and the ER reporter Sec63-mCherry in wild-type cells at the PDS phase. The arrowheads point to the Lro1-GFP punctum at the nuclear membrane. The outlines of cells are depicted. (D) Inhibition of rDNA transcription is not sufficient for stable targeting of Lro1 to the nucleolar-associated membrane. Top panel: the anchor away strain expressing an FRB-tagged version of the RNA PolI subunit RPA135 (strain SS2837), together with the parental untagged strain, were spotted on YEPD plates with or without rapamycin. Lower panel: the RPA135-FRB strain expressing Lro1-GFP were grown exponentially, rapamycin was added for the indicated times and cells were imaged live. Note that RNA PolI association with rDNA is reduced to 80% after 60 min rapamycin addition (Albert et al., 2016). Scale bars, 5 microns. Related to Figure 1.

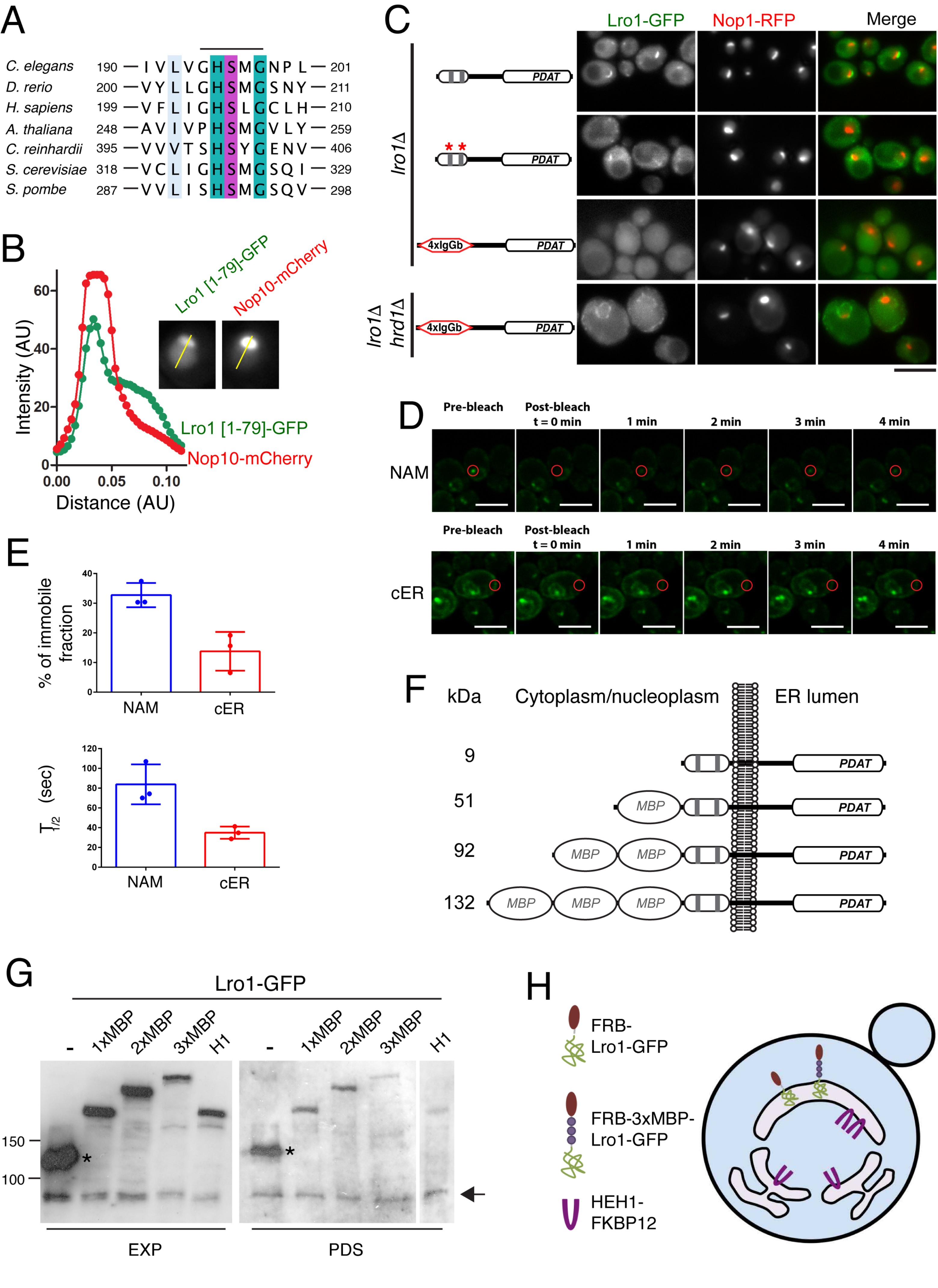

Figure S2. Related to Figure 2

Figure S2: Determinants and dynamics of Lro1 targeting to the inner nuclear membrane. (A) Alignment of the predicted catalytic GHSXG catalytic motif of Lro1 with other fungal, plant PDATs or metazoan LCATs that share a similar catalytic domain. (B) Pixel intensity profiles for Lro1[1-79]-GFP (green) and Nop10-mCherry (red) for the nucleus selected in the inset in Figure 2B; AU, arbitrary units. (C) Representative examples from the quantification shown in Figure 2C. (D) Typical FRAP of Lro1-GFP, expressed under the control of the *NOP1* promoter, at the nucleolar-associated membrane (NAM) or the cortical ER (cER). (E) Immobile fraction (top) and half-life (bottom) from measurements in 21 cells from three different experiments. (F) Schematic of the MBP fusions used; left column indicates the molecular mass (kDa) of the extralumenal domain appended. (G) A strain lacking all enzymes responsible for neutral lipid production (*dga1*Δ *lro1*Δ *are1*Δ *are2*Δ; 4Δ) was transformed with *CEN/URA3* plasmids expressing GFP fusions of *LRO1, LRO1-1xMBP -2xMBP, 3x-MBP and H1-LRO1*, all under the control of the *NOP1* promoter. Cells were grown to the exponential or PDS phases and equal amounts of cells were lysed and analysed (1.8 OD_{600} per lane) by western blot using an anti-GFP antibody. The star indicates the wild-type Lro1-GFP fusion in the exponential and PDS phases. The arrow denotes an antibody cross-reacting band. (H) Schematic of the anchor away assay used and the Lro1 fusions expressed; see text for details. Scale bars, 5 microns. Related to Figure 2.

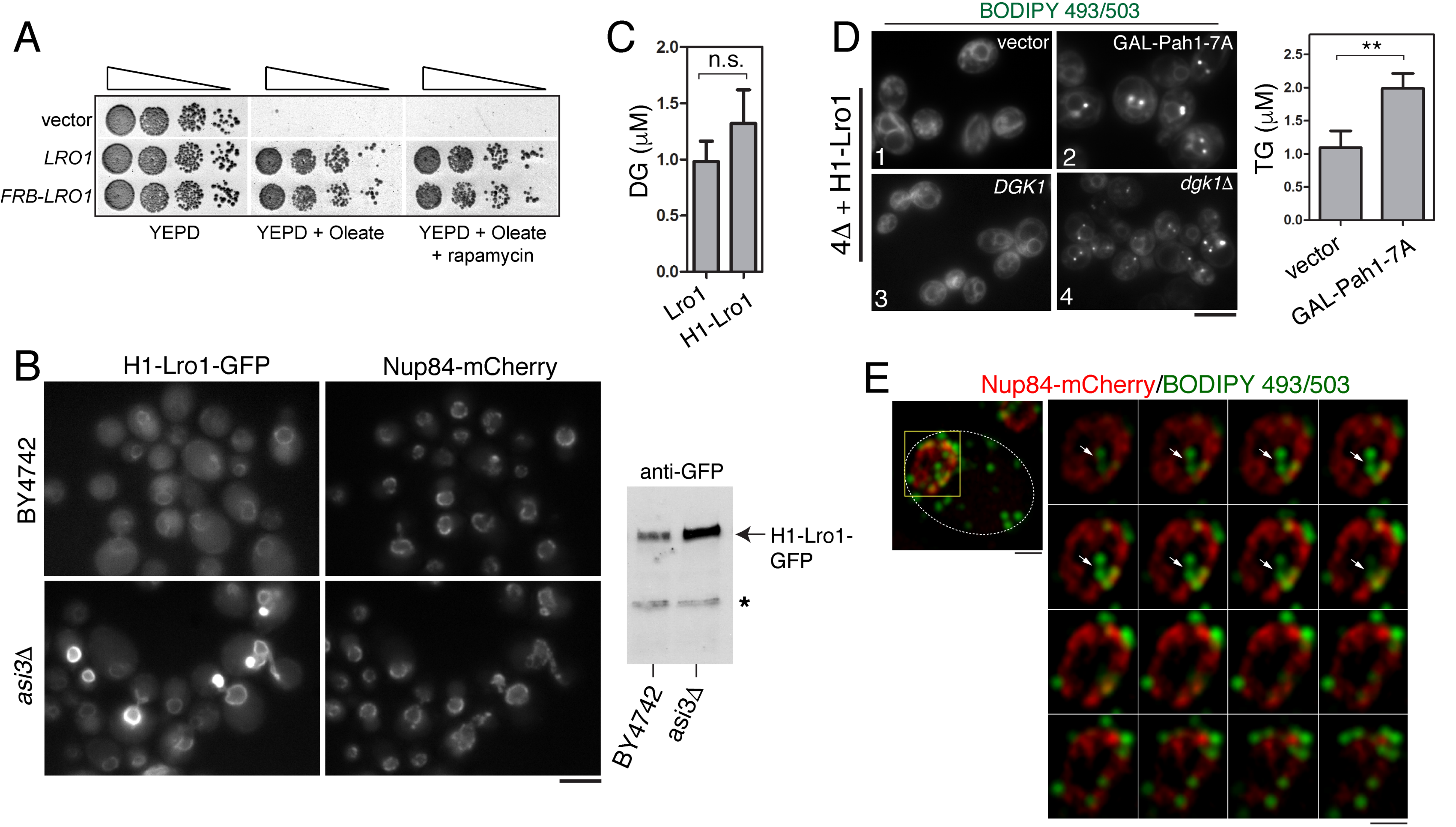

Figure S3. Related to Figures 4 and 5

Figure S3. Lro1 targeted constitutively at the INM is catalytically active. (A) A *dga1*Δ *lro1*Δ anchor away strain carrying the INM anchor (*HEH1-FKBP12*) and expressing the indicated fusions, were spotted on YEPD plates containing the denoted supplements. Cells were grown for two days at 30C. (B) H1-Lro1-GFP levels at the nuclear membrane increase in the *asi3*Δ mutant. Left panel: The indicated strains, expressing H1-Lro1-GFP and a Nup84-mCherry reporter, were grown to the PDS phase and imaged live. Right panel: samples from the same strains were lysed and analysed by western blot with an anti-GFP antibody as described under STAR Methods. The star denotes an antibody cross-reacting band. Scale bars: 5 microns. (C) Lipidomic quantification of cellular DG levels of the strains shown in Figure 5E, panels 1 and 2. DG levels shown are relative to internal DG standards of known concentration. Values are means from three independent cultures per strain. (D) 4Δ expressing H1-Lro1 and carrying an empty vector (panel 1) or the *GAL-PAH1-7A* plasmid (panel 2) were transferred to galactose-containing medium for 3 h and stained with BODIPY 493/503; right panel: samples from the cells shown in panels 1 and 2 were processed for lipidomic analysis as in C; values shown are means from three independent cultures per strain. Lower panels: exponentially growing 4Δ (panel 3) or 4Δ *dgk1*Δ (panel 4) cells expressing H1-Lro1 were stained with BODIPY 493/503. (E) 4Δ cells expressing Lro1 and Nup84-mCherry were grown to the PDS phase, stained with BODIPY 493/503, and imaged live using Zeiss LSM880 confocal microscope equipped with an Airyscan unit, as described in STAR Methods, at 0.18 μm axial resolution, and 0.2 μm step slices with 50% overlap. The arrow points to a representative intranuclear LD. Scale bars in B and D: 5 microns; in E: 1 micron. **, $p<0.01$; ns, not significant. Related to Figures 4 and 5.
